# Comparative Efficacy of Interventions for Burnout in Physician Residents: A Systematic Review and Network Meta-Analysis of Randomized Controlled Trials

**DOI:** 10.31662/jmaj.2026-0032

**Published:** 2026-05-29

**Authors:** Yukiyoshi Sumi, Kenshiro Tabata, Yosuke Hatakeyama, Toshio Morizane, Hatoko Sasaki, Fujimi Kawai, Noriko Kojimahara

**Affiliations:** 1 Department of Psychiatry, Shiga University of Medical Science, Shiga, Japan; 2 Department of Somnology, Tokyo Medical University, Tokyo, Japan; 3 Department of Pediatrics, Fukushima Medical University School of Medicine, Fukushima, Japan; 4 Department of Social Medicine, Toho University School of Medicine, Tokyo, Japan; 5 Section of Epidemiology, Shizuoka Graduate University of Public Health, Shizuoka, Japan

**Keywords:** resident, burnout, reflective interventions, systematic review, network meta-analysis, occupational health, randomized controlled trial

## Abstract

**Background:**

Burnout during residency is common and is associated with adverse outcomes for clinicians and patients; however, the comparative efficacy of intervention approaches remains unclear. We aimed to compare the efficacy of different categories of interventions aimed at reducing burnout among physician residents.

**Methods:**

We conducted a systematic review of randomized controlled trials (RCTs) in physician residents (International Prospective Register of Systematic Reviews [PROSPERO]: CRD42025610352). Searches were conducted from February 19 to 26, 2025 in PubMed, Cochrane CENTRAL, PsycINFO, Web of Science, and Ichushi-Web.

Interventions were classified into six categories: coaching, reflective intervention, intensive care unit short shift schedule, mind-body intervention, skills-based stress management, and information provision. Outcomes were emotional exhaustion (EE), depersonalization (DP), reduced efficacy (RE), and global burnout (GB). We performed a Bayesian random-effects network meta-analysis and reported standardized mean differences (SMDs) with 95% credible intervals (CrI); negative SMD values indicate improvement. Interventions were ranked using the surface under the cumulative ranking curve (SUCRA). Uncertainty was assessed using posterior effect estimates with 95% CrI and ranking probabilities. Certainty was assessed using Grading of Recommendations Assessment, Development and Evaluation (GRADE) summary of findings tables.

**Results:**

A total of 20 RCTs (n = 2,616) were included; risk of bias was mostly rated as some concerns (5% low, 60% some concerns, and 35% high risk). Effects were generally small, and estimates were imprecise. By SUCRA, reflective interventions ranked highest for EE and DP (EE: SMD −0.515 [95% CrI, −1.068 to 0.041]; DP: SMD −0.432 [−0.988 to 0.133]), whereas coaching ranked highest for RE and GB (RE: SMD −0.142 [−0.538 to 0.262]; GB: SMD −0.451 [−1.180 to 0.195]). Certainty was predominantly low to very low.

**Conclusions:**

The comparative superiority of any single intervention category remains unclear. Reflective interventions, which support guided reflection on clinical work, and coaching showed comparatively favorable signals for some burnout domains, but estimates were imprecise and of low certainty. Findings may inform cautious prioritization and highlight the need for pragmatic, adequately powered trials.

## Introduction

Burnout, recognized by the World Health Organization as an occupational phenomenon, is a common and serious health concern in healthcare systems worldwide ^(1), (2)^. Based on Maslach’s framework, burnout includes three interrelated domains: emotional exhaustion (EE), depersonalization (DP), and personal accomplishment (PA) ^(3), (4), (5), (6)^. In this study, PA is operationalized as reduced efficacy (RE) to harmonize effect directions across instruments (see Materials and Methods).

Burnout adversely affects clinicians―contributing to mood symptoms, family conflict, erosion of professional identity, and early career departure―and patients, with associations to medical errors, complications, longer hospital stays, legal risks, and lower satisfaction with care ^(4), (7), (8), (9), (10)^. At the system level, the economic burden is substantial (e.g., US $4.6 billion annually in the United States) ^(11)^. Among physicians in training, residency is widely regarded as one of the most demanding phases of medical education. An estimated one-third to half of residents experience burnout across countries and specialties ^(12), (13)^, and meta-analytic summaries indicate a 28.8% prevalence of depressive symptoms in this population ^(14)^. Although burnout is conceptually distinct from major depressive disorder, symptoms can overlap and the conditions may co-occur in physician residents. This review focuses on burnout domains (EE/DP/RE/global burnout [GB]) as intervention targets while acknowledging related mental health burdens. Burnout in residency has also been linked to attrition, medical error, and, in a concerning minority, suicidality ^(8), (10), (15), (16), (17)^. Together, these concerns highlight burnout as an important target for early recognition and support during residency training.

To alleviate burnout, programs have used a wide range of strategies, from individual-focused approaches (e.g., mindfulness and coaching) to organizational approaches (e.g., duty-hour redesign). Individual-focused approaches can also include reflective strategies that help residents process the emotional and interpersonal demands of clinical work, and such reflection-oriented support has been highlighted as a potentially valuable component of resident well-being efforts ^(18), (19)^. However, trial results have varied across studies, and prior studies typically report small pooled effects with uncertain practical significance ^(7)^. Moreover, structural reforms alone (e.g., duty-hour limits) can be offset by work compression ^(19)^, suggesting that durable progress likely requires bundled, context-sensitive approaches that pair organizational design with individual support. Although the evidence base has expanded, important research gaps remain, such as the non-randomized designs, reliance on unblinded self-reported outcomes, and overall high risk of bias, leading to very low certainty ^(7)^. In addition, pairwise meta-analyses cannot compare the multiplicity of strategies simultaneously or generate a defensible ranking of options when head-to-head trials are scarce. A network meta-analysis (NMA) can address this gap by estimating comparative efficacy across competing intervention categories.

Accordingly, we conducted a systematic review restricted to randomized controlled trials (RCTs) and applied an NMA to compare six intervention categories (e.g., mind-body intervention, coaching, information provision, intensive care unit (ICU) short shift, reflective intervention, and skills-based stress management; details are described in the Materials and Methods section) across four burnout outcomes (EE, DP, RE, and GB). Our aim was to identify strategies showing relatively favorable signals and to provide evidence-informed guidance for prioritizing resources in physician resident well-being initiatives. By integrating randomized evidence, we aim to inform decisions about where to invest to protect physician resident well-being.

## Materials and Methods

### Protocol and registration

The protocol was registered in the International Prospective Register of Systematic Reviews (PROSPERO Center for Reviews and Dissemination, University of York, York, United Kingdom) under CRD42025610352 before beginning the study search process. After registration, several clarifications were made before data extraction to make the NMA clinically meaningful: we broadened the intervention scope beyond mind-body to six categories, allowed active control comparators, harmonized burnout measures into four burnout scales, and adopted end-of-follow-up as the primary time point. All amendments and rationales are summarized in Table S1.

### Study selection

Inclusion criteria were as follows: (i) study design: RCTs; (ii) participants: physician residents in any specialty; (iii) interventions: individual- or organizational-level psychological interventions (individual: mindfulness training, meditation sessions, self-care courses, and psychological workshops; organizational: recreational opportunities, healthy food options, protected rest days following shifts, shift schedule adjustments, and shift duration modifications [e.g., short shift models]); (iv) comparators: wait-list control, pre-intervention, or active control; (v) outcomes: burnout assessed using validated scales (e.g., the Maslach Burnout Inventory [MBI] ^(3), (4), (5), (6)^); and (vi) time point: end of follow-up.

Exclusion criteria were as follows: (i) non-randomized studies; (ii) studies not enrolling physician residents; and (iii) studies not reporting any burnout outcome measure.

### Study selection―use of active learning (ASReview)

Title/abstract screening was performed using an active-learning workflow in ASReview LAB (version 1.6.3) ^(20)^. We applied a prespecified stopping rule (termination after ≥ max [1% of the corpus or 25] consecutive excludes) ^(21)^. To mitigate false-negative risk, we verified that sentinel trials from prior reviews were retrieved and included ^(7)^. Further details are provided in Supplementary Methods.

### Search strategy

This study was conducted according to the Preferred Reporting Items for Systematic Reviews and Meta-Analyses for NMA (PRISMA-NMA) guidelines ^(22)^. We searched the following electronic databases: PubMed, PsycINFO OvidSP, Cochrane Central Register of Controlled Trials (CENTRAL) in the Cochrane Library, Web of Science, and Ichushi-Web. All databases were searched for terms related to residents/residency and RCT during February 19-26, 2025 (Table S2). One author (F.K.) searched the databases, and two authors (Y.S. and K.T.) independently evaluated the eligibility of the articles and extracted the data.

### Intervention category

The protocol framed interventions as individual or organizational. After confirming study eligibility, we refined this high-level scheme before quantitative grouping and without access to effect estimates, so that nodes reflected the actual intervention landscape. Two reviewers classified each trial independently; disagreements were resolved by consensus. Interventions were mapped to six prespecified categories used for network nodes: mind-body intervention (mbi) (e.g., mindfulness/meditation/self-care workshops); coaching (coa); information provision (ifp); ICU short shift (iss) (e.g., schedule redesign and shorter/safer shifts); reflective intervention (rfi) (e.g., facilitator-led group reflection such as debriefing sessions or Balint-type groups, focusing on emotional and interpersonal aspects of clinical work); and skills-based stress management (ssm) (e.g., coping/problem-solving, pacing, and cognitive/behavioral skills). Variations in delivery mode (group vs individual), contact format (in-person vs online), and minor content differences were treated as differences within the same intervention category.

Intervention categories were refined after study selection but were finalized before data extraction to enable a clinically interpretable network. The taxonomy was agreed upon by all authors with input from a board-certified psychiatrist (Y.S.), and included trials were assigned to one of the six categories. When interventions included multiple components, categorization was based on the dominant component described as the primary intervention target.

### Data extraction and outcome measures

We extracted study characteristics (country, specialty/department, sample size per arm, sex, and age), intervention details (content, contact hours/sessions, and duration), comparator type, and pre- and post-outcome data. When multiple post-intervention assessments were reported, we selected the data at the end of follow-up.

For burnout outcomes, when studies reported only medians and interquartile ranges, confidence intervals, or missing standard deviations (SDs), we applied simple transformations to derive means and SDs.

### Outcome

Burnout measures were mapped to four harmonized domains to enable grouping across instruments: EE, DP, RE, and GB.

### Instruments encountered

The MBI (full version) and the short forms MBI-9 and MBI-5 ^(3), (4), (5), (6)^; Professional Fulfillment Index (PFI) with subscales Work Exhaustion, Interpersonal Disengagement, and Professional Fulfillment ^(23)^; Professional Quality of Life (ProQOL) Burnout subscale ^(24)^; and Copenhagen Burnout Inventory (CBI) (total and personal/work-related subscales) ^(25)^.

### Outcome selection and harmonization

We selected EE, DP, and RE as the core burnout domains, reflecting the established Maslach framework (EE/DP/personal accomplishment) ^(3), (4), (5), (6)^. RE was used to operationalize the inverse of personal accomplishment (i.e., lower personal accomplishment corresponds to higher RE), to maintain conceptual consistency across domains. We also included GB because overall/composite burnout scores are sometimes used as the primary burnout outcome in literature. We considered all four domains (EE/DP/RE/GB) clinically important and did not prioritize a single domain as primary. We harmonized all scales so that higher values reflect worse status; therefore, negative standardized mean differences (SMD) values indicate improvement across all outcomes.

1. EE―for which higher scores indicate worse status―was captured with the MBI-EE subscale (full or short forms) ^(3), (4), (5), (6)^, with the PFI work exhaustion subscale ^(23)^ and the CBI total or personal/work-related subscales ^(25)^ accepted as conceptually equivalent measures given their exhaustion-dominant content ^(26), (27)^.

2. DP―also higher scores indicate worse status―was captured primarily with MBI-DP (full or short forms) ^(3), (4), (5), (6)^ and with PFI interpersonal disengagement subscale ^(23)^.

3. RE consolidated “personal-accomplishment/efficacy-type” measures; we reverse-coded MBI-PA (full or short forms) ^(3), (4), (5), (6)^ and PFI professional fulfillment subscale ^(23)^, so that higher values reflect reduced efficacy (i.e., worse).

4. GB was reserved for overall indices not reducible to a single facet, such as ProQOL burnout scale ^(24)^; we did not derive GB by algebraically combining EE and DP to avoid double counting.

### Risk of bias

We assessed risk of bias using the Cochrane risk-of-bias tool for randomized trials version 2 (RoB 2), across five domains: (D1) randomization process; (D2) deviations from intended interventions; (D3) missing outcome data; (D4) measurement of the outcome; and (D5) selection of the reported result ^(28)^. Because all outcomes were based on self-reported burnout questionnaires, we assessed risk of bias at the study level and applied the same RoB 2 judgments to all burnout outcomes reported within each trial. Two reviewers independently judged each domain as “low risk,” “some concerns,” or “high risk;” discrepancies were resolved by consensus.

To handle trials with incomplete reporting consistently, we specified the following operational rules. When allocation concealment and/or sequence generation was not clearly described, D1 (“randomization process”) was rated as “some concerns” due to unclear risk rather than confirmed methodological flaws. Overall RoB 2 judgments were then derived using a specific algorithm: trials with “some concerns” in D1 plus “some concerns” in no more than two additional domains were classified overall as “some concerns”; trials with “some concerns” in three or more domains excluding D1 or any “high risk” in any domain were classified overall as “high risk.” In descriptive summaries (Figure S1), each study contributed equal weight. Detailed, per-study domain judgments are provided in Figure S2.

### Publication bias

For each outcome network, we inspected comparison-adjusted funnel plots and performed Egger’s regression and Begg’s rank-correlation tests for small-study effects. Tests were run at the outcome level pooling contrasts against the common control, consistent with a star-shaped network.

### Statistical analysis

#### Effect measures and NMA

For each outcome―EE, DP, RE, and GB―we calculated the mean change from baseline to follow-up for each intervention arm. To enable comparison across studies using different measurement scales, we standardized the change scores using the pooled SD of each study. The standardized mean change was defined as:

Standardized Mean Change = (Mean*_t2_* − Mean*_t1_*)/pooled SD,

where Mean*_t2_* and Mean*_t1_* refer to the group means at follow-up and baseline, respectively.

In the NMA, eligible comparator arms (wait list, pre-intervention, and active controls) were coded as a single common “control” node. A Bayesian random-effects NMA was performed separately for each outcome using the *gemtc* package in R ^(29)^. Markov chain Monte Carlo simulation was conducted with the following settings: 4 chains, 50,000 iterations, a burn-in of 10,000 iterations, and a thinning interval of 1. Convergence was evaluated using the default diagnostics provided by *gemtc*, including the Gelman-Rubin statistic, without imposing additional manual thresholds. Vague prior distributions were used for all model parameters. Treatment effects were assigned normal priors with mean 0 and variance 10,000, and between-study heterogeneity was modeled using a uniform prior on the standard deviation (Uniform[0, 5]).

Because the available evidence primarily consisted of comparisons between several individual interventions and a control group, the network structure did not allow for meaningful assessment of global or local inconsistency (e.g., design-by-treatment interaction models or node-splitting), and inconsistency analyses were therefore not performed.

For each network, we estimated SMDs with corresponding 95% credible intervals (CrI). Between-study heterogeneity was assessed using the I^2^ statistic, derived from the random-effects variance.

The statistical significance level was set at p < 0.05, and all analyses were conducted using R version 4.4.3 (R Foundation for Statistical Computing).

### Transitivity

We assumed the transitivity condition, whereby trials comparing different active interventions with a common control were considered sufficiently similar with respect to resident characteristics and trial context. Descriptive characteristics of specialty, country, intervention intensity, and follow-up length are summarized in Table 1 to allow readers to appraise this assumption.

**Table 1.** Characteristics of Included Studies.

	Study (author, year)	Country	Specialty / Department	Group	Population, N (% female)	Age (mean ± SD), years	Intervention content	Total intervention, hours	Comparator	Outcome timepoints, weeks (*1)
**Mind-body intervention: 9 studies**										
	Boden et al., 2023 ^(33)^	US	Orthopedic Surgery	Control	12 (33.0%)	30.6 ± 2.8	Provided 2-mo access to “Headspace” (mindfulness app)	Not reported	Wait list	9
				Intervention	12 (42.0%)	31.0 ± 2.8				
	Fendel et al., 2021 ^(34)^	Germany	Not specified	Control	71 (66.2%)	31.0 ± 3.5	Mindfulness-Based Stress Reduction (MBSR)-based program: 8 weekly 135-min guided group sessions + one 6-h silent retreat; 4-mo maintenance with 3 monthly booster sessions; self-care focus (theory, formal practice, group inquiry, clinical integration, and brief home tasks).	34	Information-only; no experiential or practice component	52
				Intervention	76 (64.5%)	31.0 ± 3.4				
	Fraiman et al., 2022 ^(35)^	US	Pediatrics	Control	146 (69.9%)	Not reported	Mindfulness Intervention for New Interns (MINdI) program: 7 ≤1-h monthly sessions/6 months + 10-min body-scan 2-week post-session; fits didactic hour; manual provided; no extra training.	8	Six interns-only 1-h social lunches without mindfulness activities	65
				Intervention	194 (78.9%)	Not reported				
	Ireland et al., 2017 ^(36)^	Australia	Emergency Medicine	Control	21 (not reported)	Not reported	10 × 1-h weekly mindfulness workshops (education + practice; adapted from MBSR, mindfulness-based cognitive therapy, and acceptance and commitment therapy).	10	Given an extra hour for break (one hour/week) for 10 weeks	10
				Intervention	23 (not reported)	Not reported				
	Lebares et al., 2021 ^(37)^	US	Not specified	Control	21 (43.0%)	28.7 ± 2.2	6 × 90-min weekly mindfulness classes: interoception, emotion regulation, and metacognition via breathing/body-scan/qigong; prescribed 20-min daily practice; optional off-site weekend “retreat hike” after week 4.	9	Weekly 90-min reading/discussion on stress management for 6 weeks	32
				Intervention	22 (50.0%)	28.8 ± 2.4				
	Loewenthal et al., 2021 ^(38)^	US	Not specified	Control	16 (93.8%)	29.1 (SD not reported)	6 × 60-min weekly yoga sessions (6 weeks); daily home practice encouraged.	6	Wait list	14
				Intervention	28 (75.0%)	29.3 (SD not reported)				
	Purdie et al., 2023 ^(39)^	US	Pediatrics and Medicine-Pediatrics	Control	39 (74.0%)	Not reported	Mindful Awareness Practices (MAPs): 6 × 2-h weekly mindfulness; session 1 in-person, sessions 2-6 via mobile-app self-study; daily 5-20 min practice; educator available via anonymous discussion board.	6 (*2)	Wait list	6
				Intervention	27 (89.0%)	Not reported				
	Taylor et al., 2020 ^(40)^	Australia	Not specified	Control	10 (60.0%)	30.0 ± 4.0	Personalized trauma-informed hatha yoga: 8 × 1-h weekly 1:1 sessions (pranayama, relaxation, and meditation) + 4-h workshop/retreat, 2 e-health video classes, audio-guided practice; 2-h homework requested (workshop attendance 6/10)	10.5	Group fitness	8
				Intervention	11 (91.0%)	30.0 ± 5.0				
	Verweij et al., 2018 ^(41)^	Netherlands	Not specified	Control	68 (85.0%)	31.0 ± 4.8	MBSR: 8 × ≥2-h weekly group sessions (evening options) + approximately 45-min daily audio-guided practice.	25 (*3)	Wait list	8
				Intervention	80 (90.0%)	31.4 ± 4.5				
**Skills-based stress management: 4 studies**										
	Axisa et al., 2019 ^(42)^	Australia	Internal Medicine and Pediatrics	Control	23 (78.0%)	Not reported	Half-day workshop: strategies for well-being and stress management with application planning.	4	Not reported	26
				Intervention	23 (70.0%)	Not reported				
	Bragard et al., 2010 ^(43)^	Belgium	Oncology	Control	47 (59.6%)	28.1 ± 2.2	30-h communication skills + 10-h stress-management training (small groups ≤7); role-play with immediate feedback; content: stressor detection, relaxation, cognitive restructuring, and time management	40	Wait list	22
				Intervention	49 (67.3%)	28.3 ± 3.0				
	Mache et al., 2018 ^(44)^	Germany	Emergency Medicine	Control	35 (62.0%)	27.3 ± 2.5	12 × 1.5-h weekly cognitive behavioral therapy/problem-solving sessions (work-focused; emotion-regulation strategies; and psychoeducation + experiential exercises/home practice)	18	Wait list	39
				Intervention	35 (68.0%)	27.1 ± 2.1				
	Martins et al., 2011 ^(45)^	Argentina	Pediatrics	Control	37 (86.4%)	27.6 ± 1.3	2 × 2.5-h monthly workshops (mental health professionals; burnout impact, risk recognition, and coping/self-care)	5	No intervention	9
				Intervention	37 (75.7%)	27.1 ± 1.3				
**Coaching: 3 studies**										
	Fainstad et al., 2022 ^(46)^	US	Not specified	Control	51 (100.0%)	29.6 ± 2.2	6-months web-based group coaching (“Better Together”): 2 calls/week + anonymous written coaching + weekly modules (videos/worksheets; goal setting, growth mindset, feedback, impostor syndrome, and perfectionism)	Not reported	No intervention	26
				Intervention	50 (100.0%)	29.1 ± 2.3				
	Huang et al., 2024 ^(47)^	US	Pediatric Surgery	Control	20 (70.0%)	35.8 ± 2.4	Online coaching (pediatric surgeons; positive-psychology-based), ≥3 sessions/9 months (voice/video calls)	10	Information-only; no experiential or practice component	26
				Intervention	20 (58.0%)	35.6 ± 2.2				
	Palamara et al., 2023 ^(48)^	US and Canada	Surgery or Surgical Subspecialty	Control	66 (100.0%)	30.7 ± 2.0	Online one-on-one coaching: 3 sessions over 9 months (45-60 min each)	9	Receiving 3 emails over 9 months (content unrelated to the intervention)	39
				Intervention	84 (100.0%)	30.3 ± 3.0				
**Reflective intervention: 2 studies**										
	Gunasingam et al., 2015 ^(49)^	Australia	Emergency, Internal Medicine, and Surgery	Control	18 (56.0%)	Not reported	4 × 1-h confidential debriefing sessions (every 2 weeks, after work; onsite; protocol-led by senior health professionals; not recorded)	4	No intervention	9
				Intervention	13 (38.0%)	Not reported				
	Huang et al., 2019 ^(50)^	China	Not specified	Control	18 (72.2%)	23.4 ± 0.9	Balint groups: 10 × 1-h weekly sessions (role-play/case discussion)	10	Wait list	39
				Intervention	18 (66.7%)	23.9 ± 0.9				
**Intensive care unit (ICU) short shift: 1 study**										
	Parshuram et al., 2015 ^(51)^	Canada	Intensive Care Unit (ICU)	Control	15 (not reported)	Not reported	12-h overnight duty (20:30-08:30), 3-4 consecutive nights then 72-h off; daytime duty 08:00-16:30 or 08:00-20:30; extra 20:30-21:00 handover; 4 weeks.	Not applicable	24-h overnight duty (08:00-08:30 next day); 24-h off post-call; 4 weeks	4
				Intervention	17 (not reported)	Not reported				
**Information provision: 1 study**										
	Dowers et al., 2017 ^(32)^	US	Emergency Medicine and Combined Emergency/Internal Medicine	Control	19 (not reported)	Not reported	Emails on exercise, burnout, relationships, and nutrition + daily ‘three good things’ journaling for 5 months	Not reported	No intervention	22
				Intervention	20 (not reported)	Not reported				

(*1) Months were converted using 1 month = 30.44 days (4.345 weeks). (*2) Considering an average attendance rate of 40% for online sessions, we estimated the total intervention dose at 6 h. (*3) Total intervention hours were estimated at 25 h, comprising 20 h of sessions plus 5 h of self-practice given low adherence. SD: standard deviation; US: United States.

### Ranking of interventions

To aid interpretation, we summarized the relative ranking of interventions using the surface under the cumulative ranking curve (SUCRA, 0%-100%), where higher values indicate a higher probability of being among the top-ranked options. SUCRA was treated as an auxiliary metric and was interpreted alongside the estimated effect sizes, rather than as a definitive ordering of “best” to “worst” interventions. Intervention rankings were summarized using SUCRA, mean ranks (lower values indicate higher rank), and the posterior probability of being ranked first (P[rank = 1]); these were considered descriptive and exploratory summaries of the posterior ranking distribution and were interpreted alongside effect estimates rather than as confirmatory evidence of superiority.

### Summary of findings (SoF) tables

We compiled an SoF table for each outcome (Table 3). Certainty of evidence was judged qualitatively (high, moderate, low, and very low) ^(30)^. Starting from high certainty for randomized trials, we downgraded the certainty by one or two levels according to the following domains and prespecified decision rules:

#### 1. Risk of bias

Based on RoB 2 domain-level and overall judgments for trials contributing to each comparison, we downgraded by one level when most information came from trials with “some concerns” and by two levels when major contributions came from trials judged “high” risk of bias.

#### 2. Inconsistency

We considered between-trial variability using the direction and spread of effects and heterogeneity statistics (I^2^). We downgraded when effects were inconsistent in magnitude or in direction without a plausible explanation.

#### 3. Indirectness

We assessed applicability to physician residents and typical residency settings. We downgraded by one level when evidence was restricted to a narrow population or setting that limited generalizability (e.g., ICU-only duty schedule interventions or studies enrolling only female physician residents).

#### 4. Imprecision

We used |SMD| of 0.2 as a pragmatic threshold for a small but potentially meaningful effect ^(31)^. We downgraded by one level when the 95% CrI crossed both 0 and ±0.2. We downgraded by two levels when the 95% CrI included both clinically meaningful improvement (≤ −0.2) and clinically meaningful worsening (≥ +0.2) or when evidence was extremely sparse (very small total sample size and/or very wide confidence intervals).

#### 5. Publication bias

We considered funnel plot asymmetry and statistical tests; we downgraded only when there was a clear suggestion of small-study effects.

Downgrading decisions for each comparison are documented in the Table 3 footnotes.

## Results

### Study search and selection

**Figure 1.**
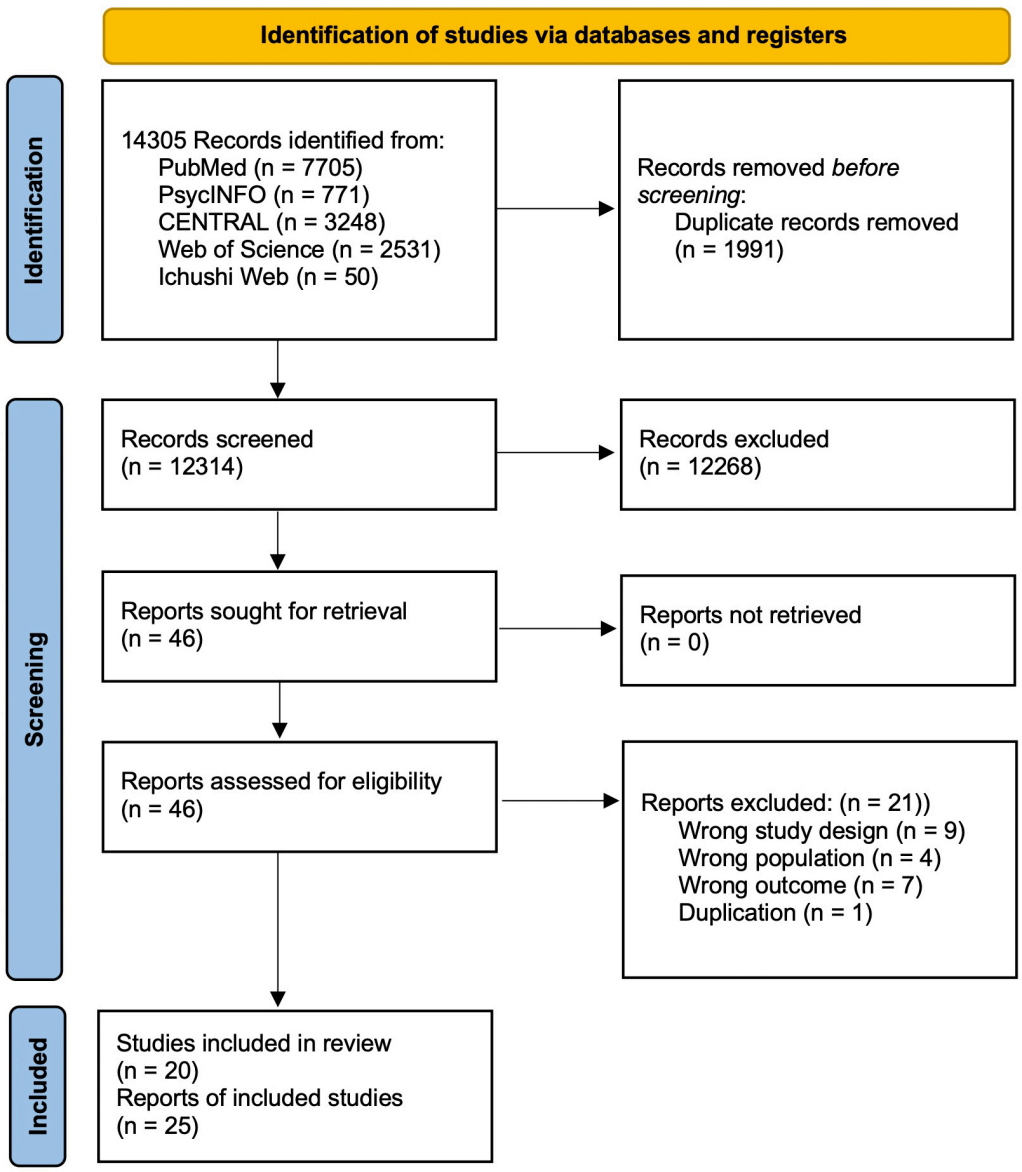
Preferred Reporting Items for Systematic Reviews and Meta-Analyses (PRISMA) flowchart.

We identified 14,305 records through database searches. After removal of duplicates, 12,314 records underwent title and abstract screening using ASReview. We applied a stopping rule of ≥124 consecutive exclusions (1% of 12,314 records); under this rule, Reviewer 1 screened 564 records and Reviewer 2 screened 479 records. Reviewer 1 judged 37 records and Reviewer 2 judged 55 records as potentially eligible; after consensus, 46 reports were retrieved for full-text review. Overall, 20 studies (reported across 25 records) met the inclusion criteria and were meta-analyzed quantitatively; 19 studies were full-length articles and one study by Dowers et al. ^(32)^ was a conference abstract. The PRISMA flow diagram is shown in Figure 1. Reports excluded after eligibility assessment (n = 21), with reasons for exclusion, are listed in Supplementary Table S3.

### Characteristics of the included RCTs

A total of 2,616 participants were included in the 20 RCTs.

Interventions were grouped into six categories: mind-body interventions (9 RCTs) ^(33), (34), (35), (36), (37), (38), (39), (40), (41)^, skills-based stress management programs (4 RCTs) ^(42), (43), (44), (45)^, coaching (3 RCTs) ^(46), (47), (48)^, reflective intervention (2 RCTs) ^(49), (50)^, ICU short shift (1 RCT) ^(51)^, and information intervention (1 RCT) ^(32)^. Countries, specialty and department settings, sample sizes, age, sex, intervention content/duration, comparators, and outcome assessment time points varied considerably across studies, as detailed in Table 1. Table S4 provides an overview of the number of studies contributing to each burnout outcome domain by intervention category. Prespecified transformations to derive SDs were required in five trials; details are provided in Table S5.

### Network geometry

**Figure 2.**
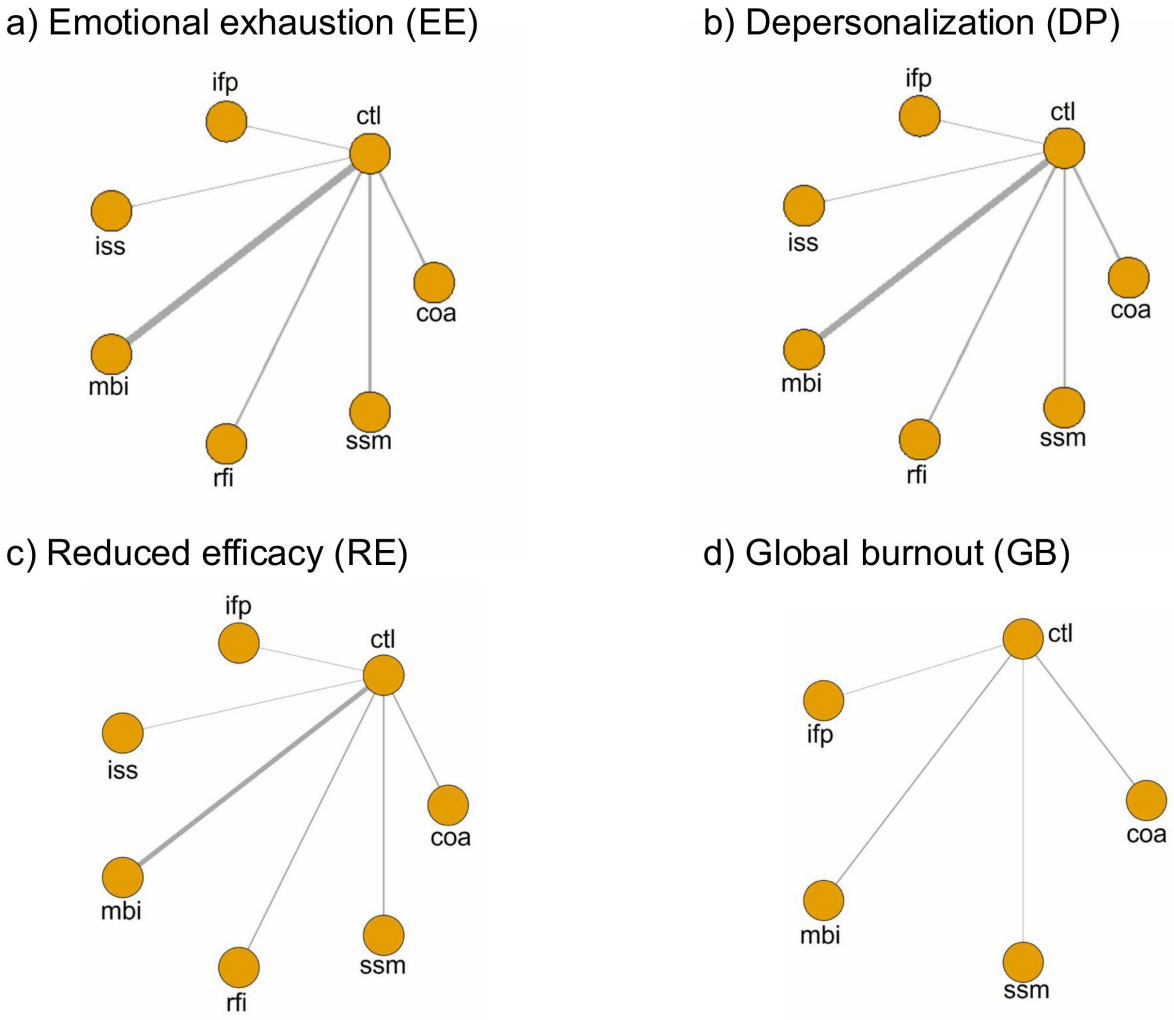
Network geometry by outcome. Outcome-specific networks for: (a) emotional exhaustion (EE), (b) depersonalization (DP), (c) reduced efficacy (RE), and (d) global burnout (GB). Nodes represent intervention categories and the common control; edges indicate available comparisons. Edge width is proportional to the number of trials per comparison. Node positions were fixed across panels to aid visual comparison. All networks are star-shaped (active interventions versus a common control). coa: coaching; ctl: control; ifp: information provision; iss: intensive care unit (ICU) short shift; mbi: mind-body intervention; rfi: reflective intervention; ssm: skills-based stress management.

Across EE, DP, RE, and GB, the networks were star-shaped with a single control node (Figure 2). Mind-body interventions contributed the largest share of trials and participants, followed by skills-based stress management, coaching and reflective intervention, ICU short shift, and information provision were less frequent. No head-to-head loops were formed.

### Risk of bias

RoB 2 summaries are shown in Figures S1 and S2. The randomization process (D1) was most often judged as “some concerns,” largely because allocation concealment was insufficiently described. Problems arose in deviations from intended interventions (D2), where many trials did not use intention-to-treat analyses and several trials reported 10%-20% attrition. By contrast, missing outcome data (D3) and measurement of the outcome (D4) were predominantly “low risk;” outcomes were validated using self-report questionnaires, administered similarly across arms. Selection of the reported result (D5) frequently showed “some concerns” or “high risk” because several trials lacked prospective registration and prespecified analytic plans, raising the possibility of selective reporting. Overall judgments were 5% “low risk,” 60% “some concerns,” and 35% “high risk” across the 20 trials.

### Outcome: EE/DP/RE/GB

**Figure 3.**
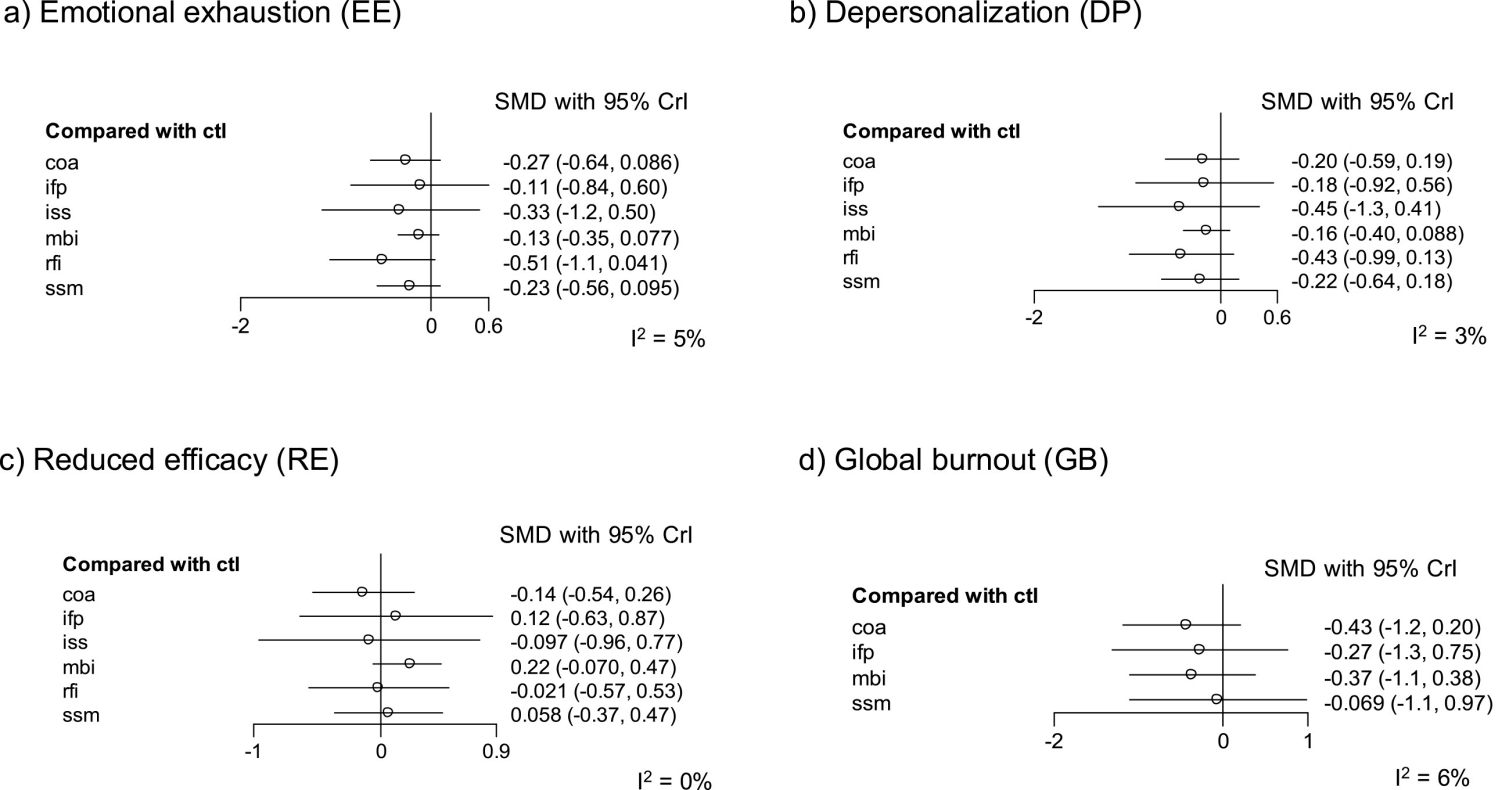
Network meta-analysis estimates versus control (forest plots). Panels show: (a) emotional exhaustion (EE), (b) depersonalization (DP), (c) reduced efficacy (RE), and (d) global burnout (GB). Points and whiskers are standardized mean differences (SMDs) with 95% credible intervals (CrI) from a Bayesian random-effects NMA; negative SMD indicates improvement (lower burnout). Panel labels display descriptive I^2^ statistic. All effects are versus the common control. coa: coaching; ctl: control; ifp: information provision; iss: intensive care unit (ICU) short shift; mbi: mind-body intervention; NMA: network meta-analysis; rfi: reflective intervention; ssm: skills-based stress management.

Figure 3 presents outcome-specific forest plots of the network estimates versus control. Across outcomes, most comparative estimates had wide CrI, often spanning both the null and the small-effect threshold (|SMD| = 0.2), indicating substantial imprecision. SUCRA-based ranking summaries are shown in Table 2, and the corresponding rankograms are shown in Figure S3.

**Table 2.** Ranking Summaries (SUCRA, Mean Rank, and P[rank = 1]) for each Intervention across Burnout Outcomes.

Intervention category	EE SUCRA	EE mean rank	EE P (rank = 1)	DP SUCRA	DP mean rank	DP P (rank = 1)	RE SUCRA	RE mean rank	RE P (rank = 1)	GB SUCRA	GB mean rank	GB P (rank = 1)
Control (ctl)	0.140	6.1	0.000	0.145	6.1	0.000	0.547	3.7	0.015	0.227	4.1	0.002
Coaching (coa)	0.603	3.3	0.083	0.500	4.0	0.056	0.733	2.6	0.272	0.721	2.1	0.344
Information provision (ifp)	0.397	4.6	0.102	0.461	4.2	0.134	0.403	4.5	0.133	0.540	2.8	0.250
ICU short shift (iss)	0.603	3.5	0.281	0.691	2.8	0.401	0.606	3.3	0.344	-	-	-
Mind-body intervention (mbi)	0.400	4.6	0.006	0.435	4.4	0.014	0.204	5.8	0.004	0.644	2.4	0.277
Reflective intervention (rfi)	0.817	2.1	0.480	0.741	2.5	0.326	0.555	3.7	0.170	-	-	-
Skills-based stress management (ssm)	0.540	3.7	0.048	0.527	3.8	0.069	0.452	4.3	0.064	0.368	3.5	0.128

SUCRA (surface under the cumulative ranking curve) values range from 0% to 100%, with higher values indicating a higher probability of ranking highly for a given outcome. Mean rank denotes the posterior expected rank (lower values indicate a higher rank). P(rank = 1) denotes the posterior probability of being ranked first. Values are based on the random-effects network meta-analysis for each domain. DP: depersonalization; EE: emotional exhaustion; GB: global burnout; ICU: intensive care unit; RE: reduced efficacy.

#### EE

Compared with control, point estimates favored reflective intervention (SMD −0.515 [95% CrI, −1.068 to 0.041]) and ICU short shift (SMD −0.331 [95% CrI, −1.153 to 0.497]); however, CrI crossed the null for all interventions, indicating uncertainty in the effects (Figure 3).

#### DP

For DP, ICU short shift (SMD −0.446 [95% CrI, −1.315 to 0.407]) and reflective intervention (SMD −0.432 [95% CrI, −0.988 to 0.133]) showed the most favorable point estimates, but all estimates remained imprecise (Figure 3).

#### RE

After direction harmonization, coaching showed a small favorable point estimate for RE (SMD −0.142 [95% CrI, −0.538 to 0.262]), with wide CrI and no clear difference from control (Figure 3).

#### GB

For GB, data were available for coaching, information provision, mind-body interventions, and skills-based stress management only. Coaching had the most favorable point estimate (SMD −0.451 [95% CrI, −1.180 to 0.195]), but the estimate was imprecise and compatible with no effect (Figure 3).

Overall, SUCRA rankings varied by outcome: reflective intervention ranked highest for EE and DP, whereas coaching ranked highest for RE and GB (Table 2, Figure S3).

### Small-study effects

For EE, DP, RE, and GB, comparison-adjusted funnel plots showed no substantive asymmetry. Egger’s and Begg’s tests were non-significant for all outcomes (Egger’s p = 0.347-0.559; Begg’s p = 0.598-1.000; Figure S4).

### SoF

Table 3 summarizes the comparative estimates and certainty of evidence for each outcome. Overall, certainty was rated as low or very low. Low certainty was observed for mind-body interventions for EE, DP, and RE; reflective intervention for EE and DP; coaching for GB; and skills-based stress management for DP, whereas the remaining comparisons were judged as very low certainty. Negative SMD values indicate improvement.

**Table 3.** Summary of Findings.

Effects of psychological and organizational interventions on burnout outcomes among physician residents						
**Population:** Physician residents in any specialty **Settings:** Hospital-based residency training programs **Intervention:** Psychological and organizational interventions, including coaching, ICU short shift models, mind-body interventions, reflective interventions, skills-based stress management, and information provision **Comparison:** Usual training conditions, wait-list control, or no intervention						
	Intervention					
Outcome	Coaching	Information provision	ICU short shift	Mind-body intervention	Reflective intervention	Skills-based stress management
Emotional exhaustion	SMD −0.274 (−0.642 to 0.086) Mean rank 3.3 n = 245 (2 studies) ⊕⊖⊖⊖ Very low ^ade^	SMD −0.118 (−0.841 to 0.599) Mean rank 4.6 n = 39 (1 study) ⊕⊖⊖⊖ Very low ^bf^	SMD −0.331 (−1.153 to 0.497) Mean rank 3.5 n = 32 (1 study) ⊕⊖⊖⊖ Very low ^cf^	SMD −0.135 (−0.348 to 0.077) Mean rank: 4.6 n = 833 (8 studies) ⊕⊕⊖⊖ Low^ ae^	SMD −0.515 (−1.068 to 0.041) Mean rank: 2.1 n = 67 (2 studies) ⊕⊕⊖⊖ Low ^ae^	SMD −0.229 (−0.565 to 0.095) Mean rank: 3.7 n = 240 (3 studies) ⊕⊖⊖⊖ Very low ^be^
Depersonalization	SMD −0.203 (−0.592 to 0.192) Mean rank 4.0 n = 245 (2 studies) ⊕⊖⊖⊖ Very low ^ade^	SMD −0.181 (−0.917 to 0.557) Mean rank 4.2 n = 39 (1 study) ⊕⊖⊖⊖ Very low ^bf^	SMD −0.446 (−1.315 to 0.407) Mean rank 2.8 n = 32 (1 study) ⊕⊖⊖⊖ Very low ^bcf^	SMD −0.155 (−0.400 to 0.088) Mean rank 4.4 n = 686 (7 studies) ⊕⊕⊖⊖ Low ^ae^	SMD −0.432 (−0.988 to 0.133) Mean rank 2.5 n = 67 (2 studies) ⊕⊕⊖⊖ Low ^ae^	SMD −0.225 (−0.644 to 0.182) Mean rank 3.8 n = 170 (2 studies) ⊕⊕⊖⊖ Low ^ae^
Reduced efficacy	SMD −0.142 (−0.538 to 0.262) Mean rank 2.6 n = 245 (2 studies) ⊕⊖⊖⊖ Very low ^adf^	SMD 0.120 (−0.634 to 0.874) Mean rank 4.5 n = 39 (1 study) ⊕⊖⊖⊖ Very low^ bf^	SMD −0.098 (−0.961 to 0.765) Mean rank 3.3 n = 32 (1 study) ⊕⊖⊖⊖ Very low ^bcf^	SMD 0.217 (−0.070 to 0.467) Mean rank 5.8 n = 643 (6 studies) ⊕⊕⊖⊖ Low ^ae^	SMD −0.019 (−0.568 to 0.533) Mean rank 3.7 n = 67 (2 studies) ⊕⊖⊖⊖ Very low ^af^	SMD 0.057 (−0.366 to 0.474) Mean rank 4.3 n = 170 (2 studies) ⊕⊖⊖⊖ Very low ^af^
Global burnout	SMD −0.451 (−1.180 to 0.195) Mean rank 2.1 n = 184 (2 studies) ⊕⊕⊖⊖ Low ^ae^	SMD −0.275 (−1.313 to 0.753) Mean rank 2.8 n = 39 (1 study) ⊕⊖⊖⊖ Very low ^bf^	―	SMD −0.368 (−1.108 to 0.377) Mean rank 2.4 n = 88 (2 studies) ⊕⊖⊖⊖ Very low ^af^	―	SMD −0.068 (−1.106 to 0.970) Mean rank 3.5 n = 46 (1 study) ⊕⊖⊖⊖ Very low ^bf^

Negative SMD values indicate improvement for burnout outcomes. Mean rank denotes the posterior expected rank (lower values indicate a higher rank). n indicates the total number of participants contributing to each intervention estimate; studies indicate the number of trials contributing to that estimate. No direct comparisons among active interventions were available; all SMDs are relative to the common control comparator. Certainty of the evidence was assessed using the GRADE approach for network meta-analysis and is presented as four levels (high, moderate, low, and very low), together with the corresponding GRADE symbols. ―, not estimable because no eligible data were available for that intervention-outcome combination. ^a^Risk of bias (−1): Downgraded one level: some concerns were common, but high risks did not dominate (RoB 2). ^b^Risk of bias (−2): Downgraded two levels: many contributing studies were at high risk of bias (RoB 2). ^c^Indirectness (−1; setting): Downgraded one level: evidence limited to ICU-only settings. ^d^Indirectness (−1; population): Downgraded one level: evidence limited to female physician residents only. ^e^Imprecision (−1): Downgraded one level: 95% CrI crossed both 0 and ±0.2. ^f^Imprecision (−2): Downgraded two levels: 95% CrI included both meaning improvement (≤ −0.2) and worsening (≥ +0.2). CrI: credible intervals; GRADE: Grading of Recommendations Assessment, Development and Evaluation; ICU: intensive care unit; SMD: standardized mean difference.

## Discussion

This NMA compared six intervention categories for physician resident burnout across four outcomes. Ranking patterns differed by outcome: reflective interventions ranked highest for EE and DP, whereas coaching ranked highest for RE and GB (Table 2, Figure S3). However, effect estimates were generally small and imprecise, and many CrI spanned both 0 and a small-effect threshold (|SMD| = 0.2). Accordingly, rankings should be read as summaries of the posterior ranking distribution rather than as proof of clinically actionable differences. Small differences in estimated effects can translate into changes in rank, particularly in a star-shaped network with limited information. We therefore emphasize effect sizes and their uncertainty as the primary results, using SUCRA, mean rank, and P(rank = 1) only to support descriptive prioritization and hypothesis generation.

### Interpretation in context

We interpret the present results as a map for relative prioritization, not a verdict for identifying a single optimal program. In settings where time and staffing are constrained, such a map can help leaders decide where to place the next unit of effort, while still weighing local feasibility, costs, and the opportunity to combine strategies. Given the predominantly low or very low certainty and overlapping CrI across interventions, we interpret these findings as comparative signals that may help frame cautious, context-specific prioritization for future evaluation, rather than as evidence supporting a single preferred intervention.

### Possible explanations for relatively favorable rankings

Reflective interventions and coaching ranked relatively favorably for some burnout domains. This may reflect differences in their primary targets: facilitated reflection (e.g., debriefing/Balint-style groups) may help physician residents process emotionally salient clinical experiences ^(49), (50)^, whereas coaching may support self-efficacy, goal setting, and boundary management ^(10), (43)^. However, these are only possible interpretations of the ranking patterns, and they were not directly tested in this review. Future trials should evaluate potential mediating mechanisms alongside burnout outcomes.

### Relation to prior evidence

These conclusions are broadly consistent with prior reviews that found modest improvements with mixed interventions, while our analysis adds value by providing comparative (albeit indirect) estimates among specific approaches. The overall pattern―small to moderate benefits with variability by outcome and context―supports selective implementation rather than wholesale adoption ^(7)^.

### Limitations

Important limitations should be considered while interpreting our findings. Because we did not search Embase or trial registries (e.g., ClinicalTrials.gov and International Clinical Trials Registry Platform [ICTRP]), eligible RCTs may have been missed, which could influence comparative estimates and rankings. We used ASReview to prioritize records for title/abstract screening and applied a prespecified stopping rule. Recent evaluations suggest that stopping rules in priority screening may miss relevant records under some conditions ^(52)^. Although we reduced this risk through independent dual screening and post-stop verification that sentinel trials from prior reviews were retrieved, some false-negative risk cannot be completely excluded.

Our network was star-shaped (all active interventions versus a common wait list/pre-intervention control), so no head-to-head loops were formed, and statistical incoherence could not be estimated. All comparisons among active interventions were informed only by indirect evidence through the common control, which may not be identical across trials. Therefore, inconsistency could not be formally assessed, and SUCRA-based rankings should be interpreted cautiously as exploratory, hypothesis-generating summaries rather than as confirmatory evidence of superiority.

Indirect comparisons in a star-shaped network rely on the transitivity assumption―that included trials are sufficiently comparable with respect to effect modifiers across intervention categories. Key study characteristics (e.g., baseline burnout severity, specialty and training environment including ICU settings, intervention format/intensity, and follow-up timing) are summarized in Table 1; however, their distribution and reporting were heterogeneous. Given the limited number of trials per intervention category and sparse overlap across categories, formal sensitivity analyses (e.g., subgroup analyses or network meta-regression by these factors) were not feasible in a meaningful way and would, in some cases, reduce to effectively single-trial contrasts. Accordingly, indirect comparisons and ranking metrics should be interpreted as comparative signals rather than as definitive evidence of superiority.

Because categorization necessarily simplifies heterogeneous, multi-component interventions, some misclassification is possible, which may attenuate or blur between-category differences.

This structure reflects our predefined PICO (patient, intervention, comparator, outcome) framework, which restricted comparators to wait list or pre-intervention conditions. Because participant blinding is inherently infeasible for these behavioral interventions, our main RoB 2 concerns instead related to allocation concealment, deviations from intended interventions (non-ITT (intention-to-treat) analyses and non-trivial attrition), and selection of the reported result (absence of prospective protocol registration) ^(28)^. These sources of bias are likely to have inflated effect estimates, particularly when analyses were not ITT. In addition, the absence of prospective registration and prespecified analysis plans raises the possibility of selective reporting, which can further exaggerate apparent benefits. Therefore, the small effects observed here should be interpreted as potentially overestimated, reinforcing our cautious interpretation of comparative differences and rankings.

### Future directions and local validation

This review did not assess implementation frameworks, costs, or resource requirements, and therefore cannot support conclusions about stepped or bundled care. Nevertheless, because burnout likely arises from both individual- and organizational-level factors, future pragmatic studies should evaluate whether multi-component approaches combining these levels are feasible, acceptable, and more effective than single-component interventions.

Rather than identifying a universally optimal program, future work should examine how comparative efficacy signals translate across different training contexts while also measuring uptake, burden, equity, and unintended consequences such as work compression ^(19)^.

Generalizability to Japanese residency programs may be limited by contextual differences in duty-hour culture, hierarchical training relationships, and the local availability of supports such as coaching or structured reflective programs. Japanese cross-sectional studies also suggest that workload and program-level factors are associated with burnout, and views on duty-hour limits remain heterogeneous ^(53), (54)^. Accordingly, local pragmatic evaluation is needed before these findings are applied to Japanese training settings.

### Conclusions

Our NMA offers a practical, though low-certainty, guide for prioritizing interventions―suggesting reflective interventions for EE/DP and coaching for RE/GB in the ranking metrics―within a broader strategy that pairs organizational redesign with individual support, delivered at the right moment.

## Article Information

### Acknowledgments

The authors thank a librarian (National Center of Neurology and Psychiatry Library) for assistance with implementing the search.

### Author Contributions

Conceptualization: Yukiyoshi Sumi, Kenshiro Tabata, Noriko Kojimahara

Methodology: Yukiyoshi Sumi, Kenshiro Tabata, Yosuke Hatakeyama, Toshio Morizane, Noriko Kojimahara

Software: Yosuke Hatakeyama, Toshio Morizane

Formal Analysis: Yosuke Hatakeyama

Investigation: Yukiyoshi Sumi, Kenshiro Tabata, Yosuke Hatakeyama

Writing-Original Draft: Yukiyoshi Sumi, Yosuke Hatakeyama

Writing-Review & Editing: Yukiyoshi Sumi, Kenshiro Tabata, Yosuke Hatakeyama, Toshio Morizane, Hatoko Sasaki, Fujimi Kawai, Noriko Kojimahara

Visualization: Yukiyoshi Sumi, Yosuke Hatakeyama

Supervision: Toshio Morizane, Hatoko Sasaki, Fujimi Kawai, Noriko Kojimahara

Project Administration: Toshio Morizane, Hatoko Sasaki, Fujimi Kawai, Noriko Kojimahara

All authors approved the final version and agreed to be accountable for all aspects of the work. Yukiyoshi Sumi and Kenshiro Tabata contributed equally to this work.

### Conflicts of Interest

None

### Approval by Institutional Review Board (IRB)

Since the online databases used in our analysis are publicly accessible, this study did not require review by an Institutional Review Board.

## Supplement

Supplementary Material

## References

[1] Burn-out an occupational phenomenon: international Classification of Diseases [Internet]. World Health Organization. 2019 May 28 [cited 2026 March 3]. Available from: https://www.who.int/news/item/28-05-2019-burn-out-an-occupational-phenomenon-international-classification-of-diseases

[2] Demerouti E, Bakker AB, Peeters MCW, et al. New directions in burnout research. Eur J Work Organ Psychol. 2021;30(5):686-91.

[3] Maslach C, Schaufeli WB, Leiter MP. Job burnout. Annu Rev Psychol. 2001;52(1):397-422. 11148311 10.1146/annurev.psych.52.1.397

[4] Maslach C, Leiter MP. Early predictors of job burnout and engagement. J Appl Psychol. 2008;93(3):498-512. 18457483 10.1037/0021-9010.93.3.498

[5] Maslach C, Leiter MP. Understanding the burnout experience: recent research and its implications for psychiatry. World Psychiatry. 2016;15(2):103-11. 27265691 10.1002/wps.20311 PMC4911781

[6] Maslach C, Jackson SE, Leiter MP. Maslach Burnout Inventory manual. 3rd ed. Palo Alto: Consulting Psychologists Press; 1996. 52 p.

[7] Kiratipaisarl W, Surawattanasakul V, Sirikul W. Individual and organizational interventions to reduce burnout in resident physicians: a systematic review and meta-analysis. BMC Med Educ. 2024;24(1):1234. 39478552 10.1186/s12909-024-06195-3 PMC11523819

[8] Zhou AY, Panagioti M, Esmail A, et al. Factors associated with burnout and stress in trainee physicians: a systematic review and meta-analysis. JAMA Netw Open. 2020;3(8):e2013761. 32809031 10.1001/jamanetworkopen.2020.13761 PMC7435345

[9] Merlo G, Rippe J. Physician burnout: a lifestyle medicine perspective. Am J Lifestyle Med. 2021;15(2):148-57. 33790702 10.1177/1559827620980420 PMC7958216

[10] Attenello FJ, Buchanan IA, Wen T, et al. Factors associated with burnout among US neurosurgery residents: a nationwide survey. J Neurosurg. 2018;129(5):1349-63. 29424650 10.3171/2017.9.JNS17996

[11] Han S, Shanafelt TD, Sinsky CA, et al. Estimating the attributable cost of physician burnout in the United States. Ann Intern Med. 2019;170(11):784-90. 31132791 10.7326/M18-1422

[12] Low ZX, Yeo KA, Sharma VK, et al. Prevalence of burnout in medical and surgical residents: a meta-analysis. Int J Environ Res Public Health. 2019;16(9):1479. 31027333 10.3390/ijerph16091479 PMC6539366

[13] Rodrigues H, Cobucci R, Oliveira A, et al. Burnout syndrome among medical residents: a systematic review and meta-analysis. PLoS One. 2018;13(11):e0206840. 30418984 10.1371/journal.pone.0206840 PMC6231624

[14] Mata DA, Ramos MA, Bansal N, et al. Prevalence of depression and depressive symptoms among resident physicians: a systematic review and meta-analysis. JAMA. 2015;314(22):2373-83. 26647259 10.1001/jama.2015.15845 PMC4866499

[15] Tawfik DS, Profit J, Morgenthaler TI, et al. Physician burnout, well-being, and work unit safety grades in relationship to reported medical errors. Mayo Clin Proc. 2018;93(11):1571-80. 30001832 10.1016/j.mayocp.2018.05.014 PMC6258067

[16] Hall LH, Johnson J, Watt I, et al. Healthcare staff wellbeing, burnout, and patient safety: a systematic review. PLoS One. 2016;11(7):e0159015. 27391946 10.1371/journal.pone.0159015 PMC4938539

[17] Ladouceur R. Resident suicide. Can Fam Physician. 2019;65(10):679. 31604726 PMC6788650

[18] Dyrbye LN, Thomas MR, Shanafelt TD. Systematic review of depression, anxiety, and other indicators of psychological distress among U.S. and Canadian medical students. Acad Med. 2006;81(4):354-73. 16565188 10.1097/00001888-200604000-00009

[19] Dyrbye L, Shanafelt T. A narrative review on burnout experienced by medical students and residents. Med Educ. 2016;50(1):132-49. 26695473 10.1111/medu.12927

[20] van de Schoot R, de Bruin J, Schram R, et al. An open source machine learning framework for efficient and transparent systematic reviews. Nat Mach Intell. 2021;3(2):125-33.

[21] Boetje J, van de Schoot R. The SAFE procedure: a practical stopping heuristic for active learning-based screening in systematic reviews and meta-analyses. Syst Rev. 2024;13(1):81. 38429798 10.1186/s13643-024-02502-7 PMC10908130

[22] Hutton B, Salanti G, Caldwell DM, et al. The PRISMA extension statement for reporting of systematic reviews incorporating network meta-analyses of health care interventions: checklist and explanations. Ann Intern Med. 2015;162(11):777-84. 26030634 10.7326/M14-2385

[23] Trockel M, Bohman B, Lesure E, et al. A brief instrument to assess both burnout and professional fulfillment in physicians: reliability and validity, including correlation with self-reported medical errors, in a sample of resident and practicing physicians. Acad Psychiatry. 2018;42(1):11-24. 29196982 10.1007/s40596-017-0849-3 PMC5794850

[24] Stamm BH. The concise ProQOL manual. 2nd ed. Pocatello: ProQOL.org; 2010. 74 p.

[25] Kristensen TS, Borritz M, Villadsen E, et al. The Copenhagen Burnout Inventory: a new tool for the assessment of burnout. Work Stress. 2005;19(3):192-207.

[26] Winwood PC, Winefield AH. Comparing two measures of burnout among dentists in Australia. Int J Stress Manag. 2004;11(3):282-9.

[27] Igawa J. Comparison of burnout scales: examination of the reliability and validity of the Japanese versions. JJ pshicho. 2025;96(4):249-59.

[28] Sterne JAC, Savovic J, Page MJ, et al. RoB 2: a revised tool for assessing risk of bias in randomised trials. BMJ. 2019;366:l4898. 31462531 10.1136/bmj.l4898

[29] van Valkenhoef G, Lu G, de Brock B, et al. Automating network meta-analysis. Res Synth Methods. 2012;3(4):285-99. 26053422 10.1002/jrsm.1054

[30] Balshem H, Helfand M, Schünemann HJ, et al. GRADE guidelines: 3. Rating the quality of evidence. J Clin Epidemiol. 2011;64(4):401-6. 21208779 10.1016/j.jclinepi.2010.07.015

[31] Schünemann HJ, Neumann I, Hultcrantz M, et al. GRADE guidance 35: update on rating imprecision for assessing contextualized certainty of evidence and making decisions. J Clin Epidemiol. 2022;150:225-42. 35934266 10.1016/j.jclinepi.2022.07.015

[32] Dowers C, Vohra T, Goyal N, et al. The effect of a resident wellness program on burnout and ITE scores. West J Emerg Med. 2017;18:S29-30.

[33] Boden LM, Rodriguez C, Kelly JD, et al. Mindfulness applications: can they serve as a stress, anxiety, and burnout reduction tool in orthopaedic surgery training? A randomized control trial. JB JS Open Access. 2023;8(3):e22.00114. 10.2106/JBJS.OA.22.00114 PMC10368388 37497194

[34] Fendel JC, Aeschbach VM, Schmidt S, et al. The impact of a tailored mindfulness-based program for resident physicians on distress and the quality of care: a randomised controlled trial. J Intern Med. 2021;290(6):1233-48. 34369618 10.1111/joim.13374

[35] Fraiman YS, Cheston CC, Cabral HJ, et al. Effect of a novel mindfulness curriculum on burnout during pediatric internship: a cluster randomized clinical trial. JAMA Pediatr. 2022;176(4):365-72. 35072694 10.1001/jamapediatrics.2021.5740 PMC8787682

[36] Ireland MJ, Clough B, Gill K, et al. A randomized controlled trial of mindfulness to reduce stress and burnout among intern medical practitioners. Med Teach. 2017;39(4):409-14. 28379084 10.1080/0142159X.2017.1294749

[37] Lebares CC, Coaston TN, Delucchi KL, et al. Enhanced stress resilience training in surgeons: iterative adaptation and biopsychosocial effects in 2 small randomized trials. Ann Surg. 2021;273(3):424-32. 32773637 10.1097/SLA.0000000000004145 PMC7863698

[38] Loewenthal J, Dyer NL, Lipsyc-Sharf M, et al. Evaluation of a yoga-based mind-body intervention for resident physicians: a randomized clinical trial. Glob Adv Health Med. 2021;10:21649561211001038. 33786209 10.1177/21649561211001038 PMC7961714

[39] Purdie DR, Federman M, Chin A, et al. Hybrid delivery of mindfulness meditation and perceived stress in pediatric resident physicians: a randomized clinical trial of in-person and digital mindfulness meditation. J Clin Psychol Med Settings. 2023;30(2):425-34. 35778655 10.1007/s10880-022-09896-3 PMC10078965

[40] Taylor J, McLean L, Richards B, et al. Personalised yoga for burnout and traumatic stress in junior doctors. Postgrad Med J. 2020;96(1136):349-57. 32300055 10.1136/postgradmedj-2019-137413

[41] Verweij H, van Ravesteijn H, van Hooff MLM, et al. Mindfulness-based stress reduction for residents: a randomized controlled trial. J Gen Intern Med. 2018;33(4):429-36. 29256091 10.1007/s11606-017-4249-x PMC5880763

[42] Axisa C, Nash L, Kelly P, et al. Burnout and distress in Australian physician trainees: evaluation of a wellbeing workshop. Australas Psychiatry. 2019;27(3):255-61. 30854868 10.1177/1039856219833793

[43] Bragard I, Etienne AM, Merckaert I, et al. Efficacy of a communication and stress management training on medical residents’ self-efficacy, stress to communicate and burnout: a randomized controlled study. J Health Psychol. 2010;15(7):1075-81. 20453053 10.1177/1359105310361992

[44] Mache S, Bernburg M, Baresi L, et al. Mental health promotion for junior physicians working in emergency medicine: evaluation of a pilot study. Eur J Emerg Med. 2018;25(3):191-8. 27879536 10.1097/MEJ.0000000000000434

[45] Martins AE, Davenport MC, Del Valle MP, et al. Impact of a brief intervention on the burnout levels of pediatric residents. J Pediatr (Rio J). 2011;87(6):493-8. 22170452 10.2223/JPED.2127

[46] Fainstad T, Mann A, Suresh K, et al. Effect of a novel online group-coaching program to reduce burnout in female resident physicians: a randomized clinical trial. JAMA Netw Open. 2022;5(5):e2210752. 35522281 10.1001/jamanetworkopen.2022.10752 PMC9077483

[47] Huang EY, Saberi RA, Palamara K, et al. Coaching program to address burnout, well-being, and professional development in pediatric surgery trainees: a randomized controlled trial. Ann Surg. 2024;280(6):938-44. 38451826 10.1097/SLA.0000000000006257

[48] Palamara K, McKinley SK, Chu JT, et al. Impact of a virtual professional development coaching program on the professional fulfillment and well-being of women surgery residents: a randomized controlled trial. Ann Surg. 2023;277(2):188-95. 35766397 10.1097/SLA.0000000000005562

[49] Gunasingam N, Burns K, Edwards J, et al. Reducing stress and burnout in junior doctors: the impact of debriefing sessions. Postgrad Med J. 2015;91(1074):182-7. 25755266 10.1136/postgradmedj-2014-132847

[50] Huang L, Harsh J, Cui H, et al. A randomized controlled trial of Balint groups to prevent burnout among residents in China. Front Psychiatry. 2019;10:957. 32116808 10.3389/fpsyt.2019.00957 PMC7026367

[51] Parshuram CS, Amaral ACKB, Ferguson ND, et al. Patient safety, resident well-being and continuity of care with different resident duty schedules in the intensive care unit: a randomized trial. CMAJ. 2015;187(5):321-9. 25667258 10.1503/cmaj.140752 PMC4361104

[52] Repke T, Tinsdeall F, Danilenko D, et al. Don’t Stop Me Now, `Cause I’m having a good time screening: evaluation of stopping methods for safe use of priority screening in systematic reviews. Don’t Stop Me Now. Cochrane Evid Synth Methods. 2026;4(1):e70068. 41583534 10.1002/cesm.70068 PMC12825451

[53] Nishimura Y, Miyoshi T, Obika M, et al. Factors related to burnout in resident physicians in Japan. Int J Med Educ. 2019;10:129-35. 31272084 10.5116/ijme.5caf.53ad PMC6766397

[54] Shikino K, Nishizaki Y, Kataoka K, et al. Optimal working hours in the 2024 physician work reform: insights from a residency program director. Adv Med Educ Pract. 2025;16:1461-8. 40861024 10.2147/AMEP.S540698 PMC12375352

