# Supplementary material for: Comparative Efficacy of Interventions for Burnout in Physician Residents: A Systematic Review and Network Meta-Analysis of Randomized Controlled Trials
Source: JMA J. 2026 May 29;9(4):705–17. doi: 10.31662/jmaj.2026-0032 (PMC13453605; doi:10.31662/jmaj.2026-0032)
Supplement: Supplementary file 1 [file 2433-3298-9-4-0705-s001.pdf]

Table S1: Protocol amendments from PROSPERO registration to the final review.

After protocol registration in PROSPERO, several modifications were made to accommodate methodological considerations required for NMA and to ensure clinically meaningful synthesis. Specifically, the final intervention classification and outcome harmonization were determined after confirming the set of eligible studies. Below we summarize the deviations from the original protocol. All amendments were made prior to data extraction and analysis.

Item	Explanations
Population	After protocol registration in PROSPERO, several modifications were made to accommodate methodological considerations required for NMA and to ensure clinically meaningful synthesis. Specifically, the final intervention classification and outcome harmonization were determined after confirming the set of eligible studies. Below we summarize the deviations from the original protocol. All amendments were made prior to data extraction and analysis.
Intervention	At registration, we planned to include only studies enrolling ≥20 participants per arm. Because several relevant trials had smaller sample sizes and excluding them would reduce generalizability and network connectivity, we removed the minimum sample-size requirement and included all eligible randomized trials regardless of group size.
Comparator	The intervention taxonomy was refined after study selection and finalized prior to data extraction to allow clinically interpretable grouping for the NMA; assignment of included trials to categories is provided in Table 1
Outcomes	Initially, burnout outcomes were limited to the MBI. We expanded eligibility to include validated burnout measures beyond MBI to avoid measurement bias and ensure comprehensive synthesis. Outcome harmonization procedures are detailed in Burnout measures and synthesis. In addition, the timing of assessment was revised. While the protocol originally specified end-of-intervention outcomes, we judged that end-of-follow-up better reflects sustained treatment effects and therefore adopted this as the primary time point.

Abbreviations: MBI, Maslach Burnout Inventory; NMA, network meta-analysis.

Table S2: Search terms for each database.

1. PubMed（February 23, 2025）

#1 Art[TW] OR Arts[TW] OR "social event*"[TW] OR autonomy[TW] OR Balint[TW] OR "brief intervention"[TW] (burnout[TW] AND prevention[TW]) OR "Career Choice"[TW] OR "clinical learning environment"[TW] OR coaching[TW] OR communication[TW] OR coping[TW] OR Counseling[TW] OR Curriculum[TW] OR debriefing[TW] OR"didactic session*"[TW] OR "discussion group*"[TW] OR distressing[TW] OR "duty*"[TW] OR "educational environment"[TW] OR "educational resource*"[TW] OR "educational session*"[TW] OR "Emotional Intelligence"[TW] OR Empathy[TW] OR Exercise[TW] OR "facilitated process group*"[TW] OR "family-oriented intervention*"[TW] OR Fitness[TW] OR "Focus Groups"[TW] OR Grit[TW] OR "Grounded Theory"[TW] OR "group concept mapping"[TW] OR "Health Behavior"[TW] OR "health checkup*"[TW] OR "Health Education"[TW] OR "Health Promotion"[TW] OR Healthy[TW] Holiday*[TW] OR Income[TW] OR "individualized meeting*"[TW]OR "Interprofessional Relations"[TW] OR Interview*[TW] OR Leadership[TW] OR "Life Style"[TW] OR Meditation[TW] OR mental[TW] OR Mentoring[TW] OR Mentor*[TW] OR Mind-Body[TW] OR mindfulness[TW] OR "Mobile Applications"[TW] OR motivation[TW] OR "Narrative Medicine"[TW] OR Neurofeedback[TW] OR Occupational Health[TW] OR Organizational Culture[TW] OR Parental Leave[TW]OR Peer Group[TW] OR activit*[TW] OR postvention[TW] OR "Practice model"[TW] OR "Program Development"[TW] OR Psychotherap*[TW] OR psychological[TW] OR "Public Health"[TW] OR schedul*[TW] OR reflection[TW] OR Resilienc*[TW] OR Role-Play[TW] OR rotation[TW] OR "Self Care"[TW] OR Self-Compassion[TW] OR shiftwork[TW] OR Sleep[TW] OR "small group*"[TW] OR "social media"[TW] OR "Social Support"[TW] OR Storytelling[TW] OR "stress management"[TW] OR "supportive faculty"[TW] OR "Team"[TW] OR sports[TW] OR teamwork[TW] OR "Resident Support"[TW] OR "Time Factors"[TW]OR "virtual reality"[TW] OR "Wearable fitness"[TW] OR ("well-being"[TW] OR wellness[TW] OR health*[TW] OR organization*[TW]) AND (program*[TW] OR intervention*[TW] OR training[TW] OR support*[TW]) OR "work hour*"[TW] OR "Working Conditions"[TW] OR "working environment"[TW] OR "working hour*"[TW] OR "Work-Life Balance"[TW] OR Workload[TW] OR Workplace[TW] OR Writing[TW] OR yoga[TW]

#2 "Death Café*"[TW] OR "proof-of-concept"[TW] OR cognitive[TW] OR napping[TW] OR Supervision[TW] OR Gatekeeper[TW] OR psychosocial[TW] OR modafinil[TW] OR feedback[TW] OR Self-Assessment*[TW] OR clerkship[TW] OR break*[TW] OR melatonin[TW] OR propranolol[TW] OR zolpidem[TW]

#3 #1 OR #2

#4 "Internship and Residency"[Mesh]

#5 (resident[TW] OR residents[TW] OR residency[TW]) AND (doctor*[tiab] OR medic*[tiab] OR physician*[tiab])

#6 (resident[TW] OR residents[TW] OR residency[TW]) NOT (aged[MH] OR child[MH] OR Infant[MH] OR Adolescent[MH] OR "Residential Facilities"[Mesh] OR "nursing home"[TIAB])

#7 intern[TW] OR interns[TW] OR internship[TW]

#8 houseman*[tw] OR housemen[tw] OR "house officer*"[tw]

#9 (junior*[tiab] OR young[tiab]OR fy1[tiab] OR fy2[tiab] OR pgy1[tiab] OR pgy2[tiab])AND (doctor*[tiab] OR medic*[tiab] OR physician*[tiab])

#10 #4 OR #5 OR #6 OR #7 OR #8 OR #9

#11 ((("randomized controlled trial"[pt] OR "controlled clinical trial"[pt] OR "randomized"[tiab] OR "placebo"[tiab]) OR ("clinical trials as topic" [mesh:noexp]) OR (randomly [tiab] OR trial [ti])) NOT (animals [mh] NOT humans [mh]))

#12 #3 AND #10 AND #11

2. PsycINFO OvidSP（February 26, 2025）

1 (Art or Arts or "social event*" or autonomy or Balint or "brief intervention" or (burnout and prevention) or "Career Choice" or "clinical learning environment" or coaching or communication or coping or Counseling or Curriculum or debriefing or "didactic session*" or "discussion group*" or distressing or duty* or "educational environment" or "educational resource*" or "educational session*" or "Emotional Intelligence" or Empathy or Exercise or "facilitated process group*" or "family-oriented intervention*" or Fitness or "Focus Groups" or Grit or "Grounded Theory" or "group concept mapping" or "Health Behavior" or "health checkup*" or "Health Education" or "Health Promotion" or "Healthy Holiday*" or Income or "individualized meeting*" or "Interprofessional Relations or Interview* or Leadership or Life Style or Meditation or mental or Mentoring or Mentor* or Mind-Body or mindfulness or Mobile Applications or motivation or Narrative Medicine or Neurofeedback or Occupational Health or Organizational Culture or Parental Leave or Peer Group or activit* or postvention or Practice model or Program Development or Psychotherap* or psychological or Public Health or schedul* or reflection or Resilienc* or Role-Play or rotation or Self Care or Self-Compassion or shiftwork or Sleep or small group* or social media or Social Support or Storytelling or stress management or supportive faculty or Team or sports or teamwork or Resident Support or Time Factors or virtual reality or Wearable fitness or (well-being or wellness or health* or organization*)) and (program* or

intervention* or training or support*)) or work hour* or Working Conditions or working environment or working hour* or Work-Life Balance or Workload or Workplace or writing or yoga").ti,ab.

2 ("Death Café*" or proof-of-concept or cognitive or napping or Supervision or Gatekeeper or psychosocial or modafinil or feedback or Self-Assessment* or clerkship or break* or melatonin or propranolol or zolpidem).ti,ab.

3 1 or 2

4 "Medical Internship".mp. [mp=ti, ab, tx, ct, hw, tc, id, ot, tm, mf]

5 ((resident or residents or residency) and (doctor* or medic* or physician*)).ti.

6 (resident or residents or residency).ti. not (exp aged/ or exp child/ or exp Infant/ or exp Adolescent/ or exp "Residential Facilities"/ or "nursing home".ti,ab.)

7 ((intern or interns or internship) and (doctor* or medic* or physician*)).ti.

8 ((houseman* or housemen or "house officer*") and (doctor* or medic* or physician*)).ti.

9 ((junior* or young*) and (doctor* or medic* or physician*)).ti.

10 4 or 5 or 6 or 7 or 8 or 9

11 Experiment Controls/ or Placebo/ or Clinical Trials/ or Randomized Controlled Trials/ or Randomized Clinical Trials/

12 Clinical trial.md.

13 (random* or sham or placebo*).ti,ab,id,hw,mf.

14 ((singl* or doubl*) adj (blind* or dumm* or mask*)).ti,ab,id,hw,mf.

15 ((tripl* or trebl*) adj (blind* or dumm* or mask*)).ti,ab,id,hw,mf.

16 (control* adj3 (study or studies or trial* or group*)).ti,ab,id,hw,mf.

17 (clinical adj3 (study or studies or trial*)).ti,ab,id,hw,mf.

18 (Nonrandom* or non random* or non-random* or quasi-random* or quasirandom*).ti,ab,id,hw,mf.

19 (phase adj6 (study or studies or trial*)).ti,ab,id,hw,mf.

20 ((crossover or cross-over) adj3 (study or studies or trial*)).ti,ab,id,hw,mf.

21 ((multicent* or multi-cent*) adj3 (study or studies or trial*)).ti,ab,id,hw,mf.

22 allocated.ti,ab,hw,mf.

23 ((open label or open-label) adj5 (study or studies or trial*)).ti,ab,id,hw,mf.

24 ((equivalence or superiority or non-inferiority or noninferiority) adj3 (study or studies or trial*)).ti,ab,id,hw,mf.

25 (pragmatic study or pragmatic studies).ti,ab,id,hw,mf.

26 ((pragmatic or practical) adj3 trial*).ti,ab,id,hw,mf.

27 ((quasiexperimental or quasi-experimental) adj3 (study or studies or trial*)).ti,ab,id,hw,mf.

28 trial.ti,id.

29 11 or 12 or 13 or 14 or 15 or 16 or 17 or 18 or 19 or 20 or 21 or 22 or 23 or 24 or 25 or 26 or 27 or 28
30 3 and 10 and 29

3. Cochrane Central Register of Controlled Trials(CENTRAL) in the Cochrane Library （February 22, 2025）

#1 (Art OR Arts OR (social NEXT event*) OR autonomy OR Balint OR (brief NEXT intervention) OR (burnout AND prevention) OR (Career NEXT Choice) OR "clinical learning environment" OR coaching OR communication OR coping OR Counseling OR Curriculum OR debriefing OR(didactic NEXT session*) OR (discussion NEXT group*) OR distressing OR duty* OR "educational environment" OR (educational NEXT resource*) OR (educational NEXT session*) OR "Emotional Intelligence" OR Empathy OR Exercise OR (facilitated NEXT process NEXT group*) OR (family-oriented NEXT intervention*) OR Fitness OR "Focus Groups" OR Grit OR "Grounded Theory" OR "group concept mapping" OR "Health Behavior" OR (health NEXT checkup*) OR "Health Education" OR "Health Promotion" OR Healthy OR Holiday* OR Income OR (individualized NEXT meeting*) OR "Interprofessional Relations" OR Interview* OR Leadership OR "Life Style" OR Meditation OR mental OR Mentoring OR Mentor* OR Mind-Body OR mindfulness OR "Mobile Applications" OR motivation OR "Narrative Medicine" OR Neurofeedback OR "Occupational Health"):ti,ab,kw
#2 ("Organizational Culture" OR "Parental Leave" OR "Peer Group" OR activit* OR postvention OR "Practice model" OR "Program Development" OR Psychotherap*):ti,ab,kw
#3 (psychological OR Public Health OR schedul* OR reflection OR Resilienc* OR Role-Play OR rotation OR Self Care OR Self-Compassion OR shiftwork OR Sleep):ti,ab,kw
#4 ("small group" OR "social media" OR "Social Support" OR Storytelling OR "stress management" OR "supportive faculty" OR Team OR sports OR teamwork):ti,ab,kw
#5 ("Resident Support" OR "Time Factors" OR "virtual reality" OR "Wearable fitness" OR (work NEXT hour*) OR "Working Conditions" OR "working environment" OR (working NEXT hour*) OR "Work-Life Balance" OR Workload OR Workplace OR Writing OR yoga):ti,ab,kw
#6 ((well-being OR wellness OR health* OR organization*) AND (program* OR intervention* OR training OR support*)):ti,ab,kw
#7 ((Death NEXT Café*) OR "proof-of-concept" OR cognitive OR napping OR Supervision OR Gatekeeper OR psychosocial OR modafinil OR feedback OR Self-Assessment* OR clerkship OR break* OR melatonin OR propranolol OR zolpidem):ti,ab,kw
#8 #1 OR # 2 OR #3 OR #4 OR #5 OR #6 OR #7
#9 MeSH descriptor: [Internship and Residency] explode all trees

#10 ((resident OR residents OR residency) AND (doctor* OR medic* OR physician*)):ti
#11 (resident OR residents OR residency):ti
#12 MeSH descriptor: [Aged] explode all trees
#13 MeSH descriptor: [Child] explode all trees
#14 MeSH descriptor: [Infant] explode all trees
#15 MeSH descriptor: [Adolescent] explode all trees
#16 MeSH descriptor: [Residential Facilities] explode all trees
#17 ("nursing home"):ti,ab,kw
#18 #12 OR #13 OR #14 OR #15 OR #16 OR #17
#19 #11 NOT #18
#20 (intern OR interns OR internship):ti
#21 (houseman* OR housemen OR (house NEXT officer*)):ti
#22 ((junior* OR young OR fy1 OR fy2 OR pgy1 OR pgy2)AND (doctor* OR medic* OR physician*)):ti
#23 #9 OR #10 OR #19 OR #20 OR #21 OR #22
#24 #8 AND #23 in Trials

4. Web of Science（February 19, 2025）

#1 TI=((Art OR Arts OR ""social event*"" OR autonomy OR Balint OR ""brief intervention"" OR (burnout AND prevention) OR ""Career Choice"" OR ""clinical learning environment"" OR coaching OR communication OR coping OR Counseling OR Curriculum OR debriefing OR ""didactic session*"" OR ""discussion group*"" OR distressing OR duty* OR ""educational environment"" OR ""educational resource*"" OR ""educational session*"" OR ""Emotional Intelligence"" OR Empathy OR Exercise OR ""facilitated process group*"" OR ""family-oriented intervention*"" OR Fitness OR ""Focus Groups"" OR Grit OR ""Grounded Theory"" OR ""group concept mapping"" OR ""Health Behavior"" OR ""health checkup*"" OR ""Health Education"" OR ""Health Promotion"" OR ""Healthy Holiday*"" OR Income OR ""individualized meeting*"" OR ""Interprofessional Relations"" OR Interview* OR Leadership OR ""Life Style"" OR Meditation OR mental OR Mentoring OR Mentor* OR Mind-Body OR mindfulness OR ""Mobile Applications"" OR motivation OR ""Narrative Medicine"" OR Neurofeedback OR ""Occupational Health"" OR ""Organizational Culture"" OR ""Parental Leave"" OR ""Peer Group"" OR activit* OR postvention OR ""Practice model"" OR ""Program Development"" OR Psychotherap* OR psychological OR ""Public Health"" OR schedul* OR reflection OR Resilienc* OR Role-Play OR rotation OR ""Self Care"" OR Self-Compassion OR shiftwork OR ""Sleep"" OR ""small group*"" OR ""social media"" OR ""Social Support"" OR Storytelling OR ""stress

management"" OR ""supportive faculty"" OR Team OR sports OR teamwork OR ""Resident Support"" OR ""Time Factors"" OR ""virtual reality"" OR ""Wearable fitness"" OR (well-being OR wellness OR health* OR organization*) AND (program* OR intervention* OR training OR support*) OR ""work hour*"" OR ""Working Conditions"" OR ""working environment"" OR ""working hour*"" OR ""Work-Life Balance"" OR Workload OR Workplace OR ""Writing"" OR yoga))

#2 TI=(""Death Café*"" OR proof-of-concept OR cognitive OR napping OR Supervision OR Gatekeeper OR psychosocial OR modafinil OR feedback OR Self-Assessment* OR clerkship OR break* OR melatonin OR propranolol OR zolpidem)

#3 #1 OR #2

#4 TI=(resident OR residents OR residency OR (doctor* OR medic* OR physician*) OR ((resident OR residents OR residency) NOT (aged OR child OR Infant OR Adolescent OR residential OR ""nursing home"")) OR intern OR interns OR internship OR houseman* OR housemen OR ""house officer*"" OR ((junior* OR young OR fy1 OR fy2 OR pgy1 OR pgy2) AND (doctor* OR medic* OR physician*)))

#5 TS=(randomised OR randomized OR randomisation OR randomization OR placebo* OR (random* AND (allocat* OR assign*)) OR (blind* AND (single OR double OR treble OR triple)))

#6 #3 AND #4 AND #5

5. Ichushi-Web（February 25, 2025）

(((("医師臨床研修"/TH) or (研修医/TA or インターン/TA or レジデント/TA or 専攻医/TA) or (若手医師/TA))) and (PT=原著論文 and RD=ランダム化比較試験,準ランダム化比較試験))

(((("Internship and Residency "/TH) or (resident/TA or intern/TA) or (“young doctor”/TA))) and (PT=Research Article and RD=Randomized Controlled Trial, Quasi-Randomized Controlled Trial)) 【Search in Japanese】

Note: For Ichushi-Web, we intentionally did not include intervention-related keywords and instead searched only resident-related terms combined with filters, in order to capture any randomized controlled trials involving physician residents regardless of the type of intervention.

Supplementary Figures

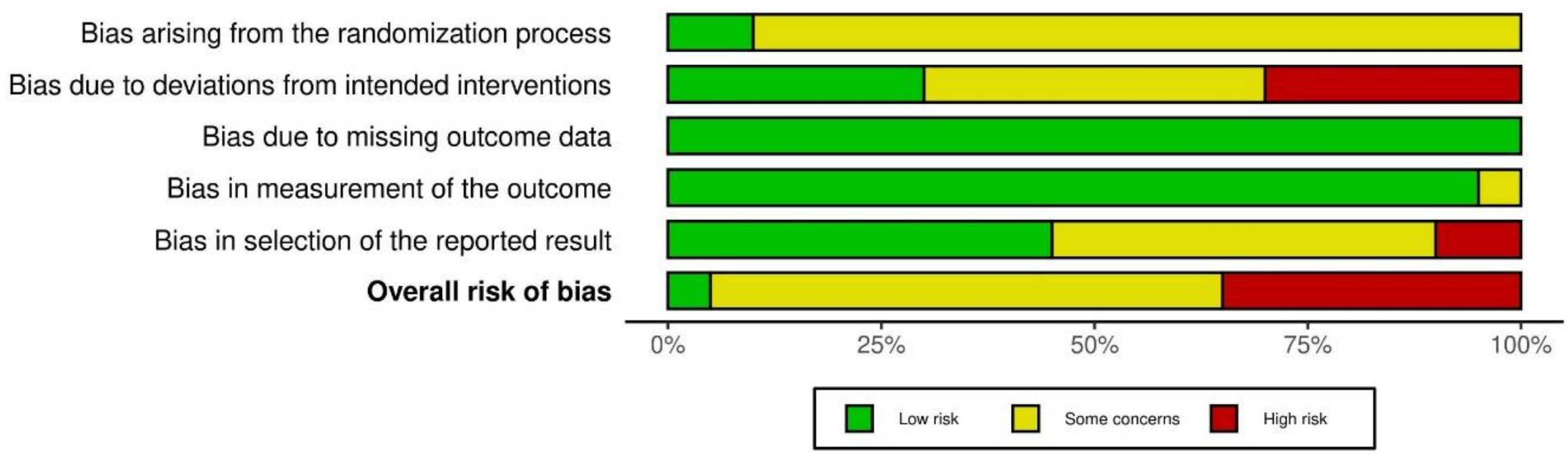

Figure S1: Risk-of-bias (RoB 2) summary across included randomized trials.

Stacked bars show the proportion of included randomized trials judged Low risk (green), Some concerns (yellow), or High risk (red) within each RoB 2 domain and for the Overall risk of bias. Each trial contributes equally (unweighted counts).

Domains: D1 Bias arising from the randomization process; D2 Bias due to deviations from intended interventions; D3 Bias due to missing outcome data; D4 Bias in measurement of the outcome; D5 Bias in selection of the reported result.

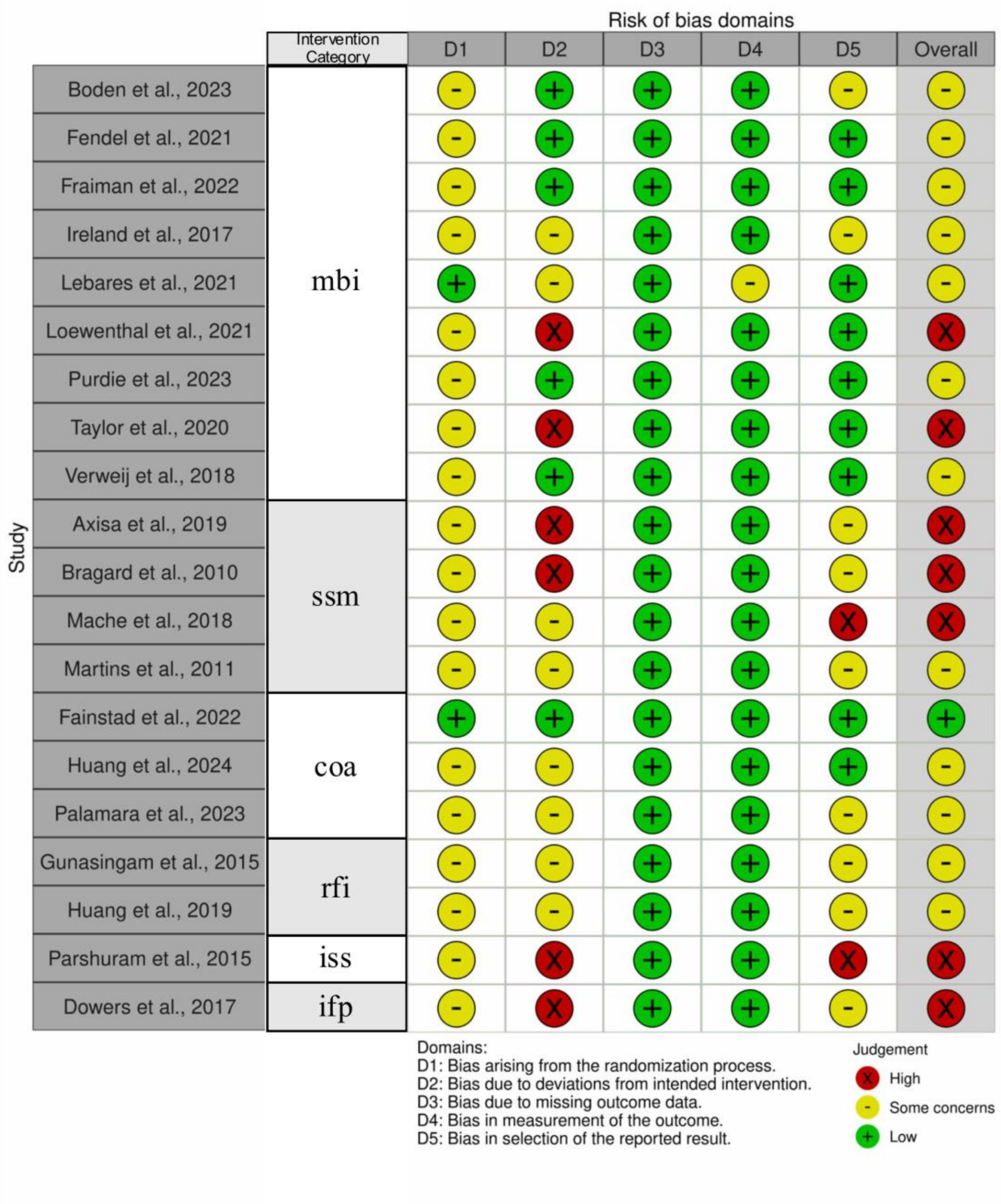

Figure S2: Risk-of-bias (RoB 2) traffic-light plot for individual trials ordered by intervention category.

Rows are grouped and sorted by intervention category; the "Intervention category" column indicates the category for each trial. Domains D1–D5 and Overall judgments follow RoB 2. Abbreviations: coa, coaching; ifp, information provision; iss, ICU short shift; mbi, mind–body intervention; rfi, reflective intervention; ssm, skills-based stress management.

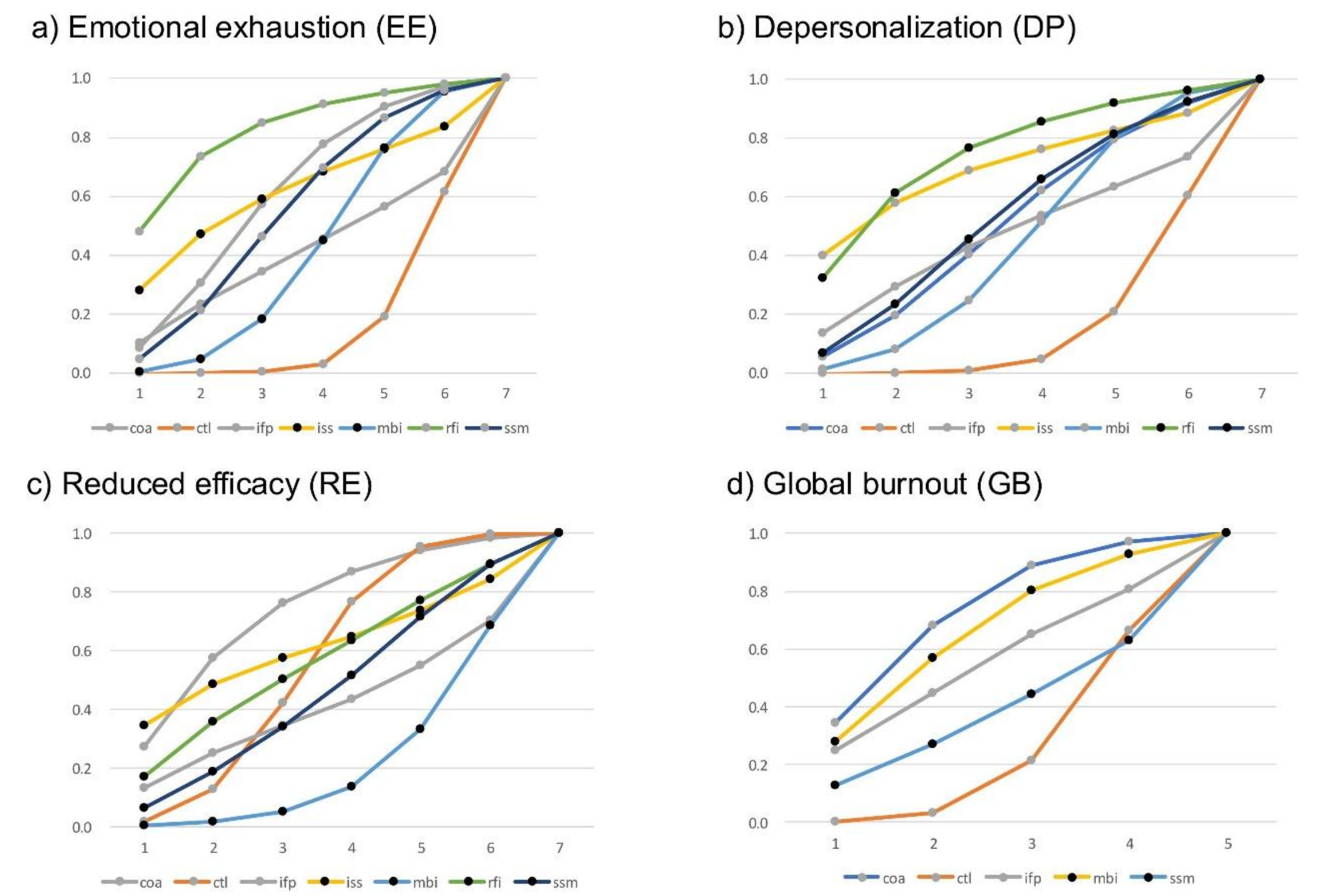

Figure S3: Rankograms and SUCRA across interventions for each burnout outcome.

For each outcome—emotional exhaustion (EE), depersonalization (DP), reduced efficacy (RE), and global burnout (GB)—curves show the cumulative probability that an intervention attains a given rank (x-axis; 1 = highest). The surface under each cumulative ranking curve (SUCRA; 0–1 or 0–100%) summarizes the ranking, with higher values indicating a greater probability of being among the top-ranked options.

Abbreviations: coa, coaching; ctl, control; ifp, information provision; iss, ICU short shift; mbi, mind–body intervention; rfi, reflective intervention; ssm, skills-based stress management.

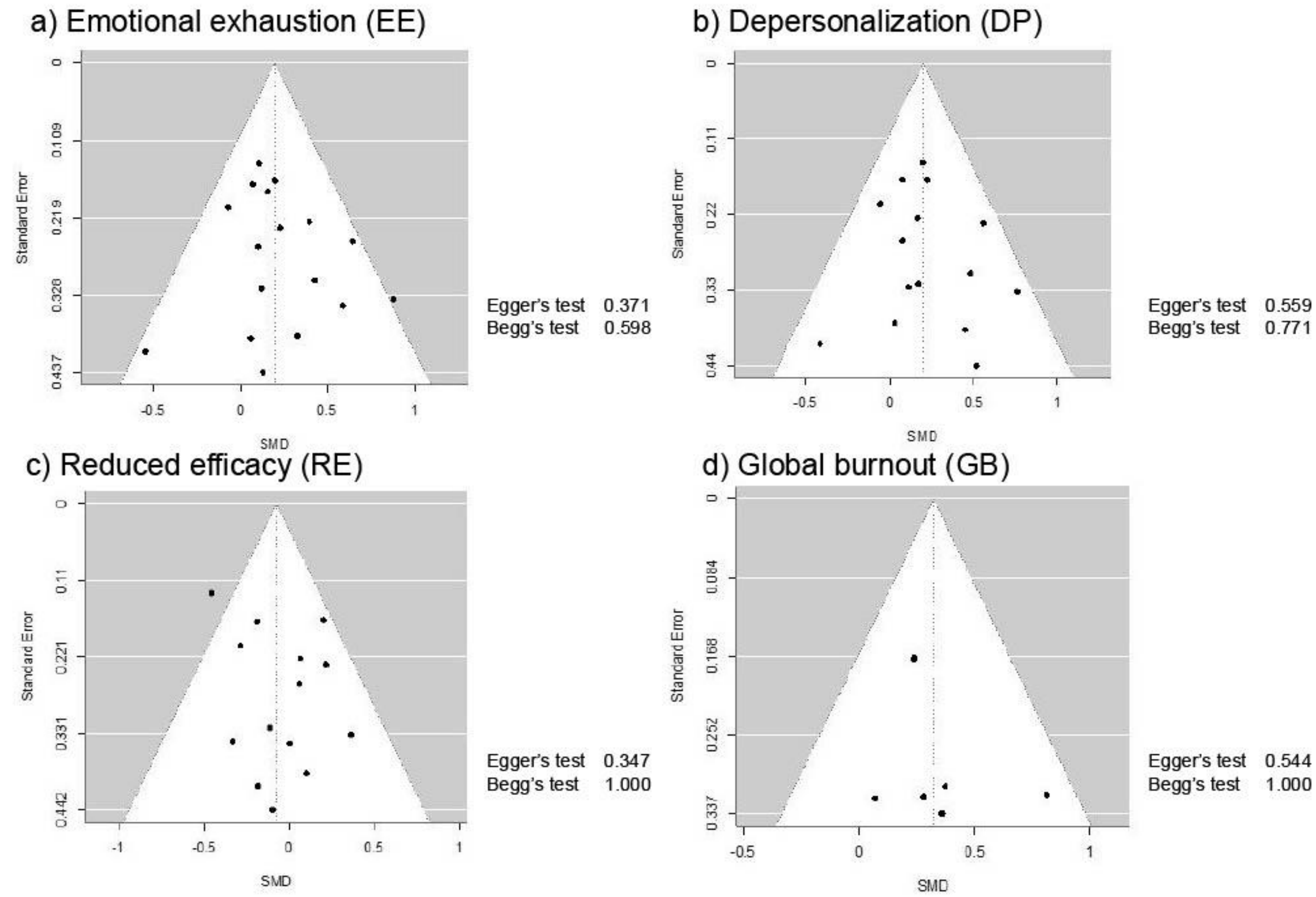

Figure S4: Funnel plots for burnout scales.

Each panel displays study-level standardized mean differences (SMD) on the x-axis versus the standard error on the y-axis; points are individual trial contrasts versus control. The vertical dashed line denotes the null (SMD = 0); sloping lines mark the 95% pseudo–confidence limits. Egger's and Begg's test p-values are shown in each panel.

Abbreviations: EE, emotional exhaustion; DP, depersonalization; RE, reduced efficacy; GB, global burnout.

Table S3: Reports excluded after eligibility assessment with reasons.

ID	Report	Reason	Note
1	Bateman ME and Chung CH and Mascarenhas E and Hammer R and Ravindran N and Panjshiri F and Mehta P and Byrne A and Lasky S and Denson R and Brown M and Halton B and Chiurco J and Ferrell S and Ruiz B and Wentowski C and Shukla I and Bauer H and Sarma A and Bhyravabhotla K and Zu Y and Peacock E and Lefante J and Epere J and Denson JL. STOPTHEBURN: A Randomized Controlled Trial of Death Cafés for Burnout Prevention in Intensive Care Unit Employees. Annals of the American Thoracic Society. 2024;21.0(11.0):1572-1582. doi:10.1513/AnnalsATS.202312-1024OC.	Wrong population	
2	Chinai, S and Bird, S and Lesperance, D and Maranda, S and Balasubramaniam, M and Haskins, B and Doherty, S and Duncan, B and Hakim, N and Boudreaux, E. Effect of an empathy curriculum on emergency medicine resident burnout and patient perception of empathy: a randomized controlled trial. Western Journal of Emergency Medicine: Integrating Emergency Care with Population Health. 2016;17(4.1):S12.	Wrong study design	
3	Cohen, B and Nirappil, J and Sokoloff, W and McCabe, ME and Broder, M. 40. CHAMILY CHALLENGE: THE IMPACT AND EFFICACY OF TEAM-BASED GAMIFICATION ON PEDIATRIC RESIDENT ENGAGEMENT. Academic Pediatrics. 2020;20(7):e20. doi:10.1016/j.acap.2020.06.061.	Wrong outcome	
4	Ferrara V and Shaholli D and Iovino A and Cavallino S and Colizzi MA and Della Rocca C and La Torre G. Visual Thinking Strategies as a Tool for Reducing Burnout and Improving Skills in Healthcare Workers: Results of a Randomized Controlled Study. Journal of clinical medicine. 2022;11.0(24.0). doi:10.3390/jcm11247501.	Wrong population	

5	Hicks MD and Braden LA and Walsh EM and Greene BJ and Grayson JW. Mobile meditation for improving quality of life, anxiety and depression among surgical residents and faculty. The Journal of laryngology and otology. 2022;136.0(11.0):1034-1038. doi:10.1017/S0022215121003091.	Wrong population
6	Khalsa, SB and Dyer, N and Loewenthal, J and Lipsyc-Sharf, M and Mehta, D and Dusek, J. A Pilot Study of a Six Week Yoga and Mindfulness-Based Program in Resident Physicians. European Journal of Integrative Medicine. 2021;48:102080. doi:10.1016/j.eujim.2021.102080.	Wrong outcome
7	Lai L and Sanatkar S and Mackinnon A and Deady M and Petrie K and Lipscomb R and Counson I and Francis-Taylor R and Dean K and Harvey S. Testing the Effectiveness of a Mobile Smartphone App Designed to Improve the Mental Health of Junior Physicians: Protocol for a Randomized Controlled Trial. JMIR research protocols. 2024;13.0:e58288. doi:10.2196/58288.	Wrong study design
8	Lebares CC and Hershberger AO and Guvva EV and Desai A and Mitchell J and Shen W and Reilly LM and Delucchi KL and O'Sullivan PS and Ascher NL and Harris HW. Feasibility of Formal Mindfulness-Based Stress-Resilience Training Among Surgery Interns: A Randomized Clinical Trial. JAMA surgery. 2018;153.0(10.0):e182734. doi:10.1001/jamasurg.2018.2734.	Wrong outcome
9	Mache S and Vitzthum K and Klapp BF and Groneberg DA. Evaluation of a Multicomponent Psychosocial Skill Training Program for Junior Physicians in Their First Year at Work: A Pilot Study. Family Medicine. 2015;47(9):693–698.	Wrong outcome
10	ClinicalTrials.gov. Does a Mindfulness Curriculum Prevent Physician Burnout During Pediatric Internship–. Identifier: NCT03148626. (First posted 2017).	Wrong study design
11	ClinicalTrials.gov. Intensive Care Unit Resident Scheduling Trial. Identifier: NCT04176094. (First posted 2019).	Wrong study design

12	ClinicalTrials.gov. Investigating the Impact of Professional Development Coaching Programs in Residents & Fellows. Identifier: NCT04883307. (First posted 2021).	Wrong study design
13	Ricker M and Brooks AJ and Chen MK and Weydert J and Locke A and Meehan EK and Cook P and Lebensohn P and Maizes V. The Feasibility and Impact of an Asynchronous Interprofessional Well-Being Course on Burnout in Health Care Professionals. HCA healthcare journal of medicine. 2024;5.0(3.0):343-351. doi:10.36518/2689-0216.1778.	Wrong population
14	Syed A and Thibodeau PS and Dieujuste N and Jones CD and Dunbar KS and Mann A and Fainstad T. Examining the Impact of Moral Injury on Burnout, Impostor Syndrome, and Low Self-Compassion Among Female Medical Trainees: A Secondary Analysis. Mayo Clinic proceedings. 2024;99.0(12.0):1905-1912. doi:10.1016/j.mayocp.2024.07.015.	Wrong study design
15	Trainor, A and Erb, E and Quinn, TM and Furfaro, D and Angoff, R and Brady, VA and Ferrera, MC and Hayes, MM. Call Vs No Call: a Pilot Study of Resident Choice in ICU Scheduling. American Journal of Respiratory and Critical Care Medicine. 2023;207(1):A4594. doi:10.1164/ajrccm-conference.2023.C23.	Wrong study design
16	Weitzman RE and Wong K and Worrall DM and Park C and McKee S and Tufts RE and Teng MS and Iloreta AM. Incorporating Virtual Reality to Improve Otolaryngology Resident Wellness: One Institution's Experience. The Laryngoscope. 2021;131.0(9.0):1972-1976. doi:10.1002/lary.29529.	Wrong study design
17	Wong, K and Worrall, D and McKee, S and Iloreta, AMC. Impact of virtual reality mindfulness training on otolaryngology resident wellness. Otolaryngology–Head and Neck Surgery. 2020;163(1S):P261. doi:10.1177/0194599820934781.	Wrong study design
18	Lockwood BJ and Gustin J and Verbeck N and Rossfeld K and Norton K and Barrett T and Potts R and Towner-Larsen R and Waterman B and Radwany S and Hritz C and Wells-Di Gregorio S and Holliday S. Training to Promote Empathic Communication in Graduate Medical Education: A Shared Learning Intervention in Internal Medicine and General Surgery. Palliative medicine reports. 2022;3.0(1.0):26-35. doi:10.1089/pmr.2021.0036.	Wrong outcome

19	Mann, A and Shah, AN and Thibodeau, PS and Dyrbye, L and Syed, A and Woodward, MA and Thurmon, K and Jones, CD and Dunbar, KS and Fainstad, T. Online Well-Being Group Coaching Program for Women Physician Trainees. JAMA NETWORK OPEN. 2023;6.0(10.0). doi:10.1001/jamanetworkopen.2023.35541.	Wrong outcome	cannot compute mean/SD
20	Ripp, JA and Fallar, R and Korenstein, D. A Randomized Controlled Trial to Decrease Job Burnout in First-Year Internal Medicine Residents Using a Facilitated Discussion Group Intervention. Journal of Graduate Medical Education. 2016;8(2):256–259. doi:10.4300/JGME-D-15-00120.1.	Wrong outcome	
21	Speckens A and Verweij H and van Ravesteijn H and Lagro-Janssen T. [Mindfulness for medical, surgical and psychiatric residents]. Tijdschrift voor psychiatrie. 2019;61(3):188–193.	Duplication	Duplicate report of an included study (Verweij et al., 2018)

Table S4: Number of studies contributing to each burnout outcome domain, by intervention category.

Intervention category	Emotional exhaustion (EE)	Depersonalization (DP)	Reduced efficacy (RE)	Global burnout (GB)
Coaching (coa)	2	2	2	2
Information provision (ifp)	1	1	1	1
ICU short shift (iss)	1	1	1	0
Mind–body intervention (mbi)	8	7	6	2
Reflective intervention (rfi)	2	2	2	0
Skills-based stress management (ssm)	3	2	2	1

Data are the number of studies that reported end-of-follow-up outcome data and were included in the network meta-analysis for each domain. A single trial may contribute to more than one outcome domain; therefore, column totals exceed the total number of included trials (n = 20). Multiple reports from the same trial were counted once.

Abbreviations:
coa, coaching; ifp, information provision; iss, ICU short shift; mbi, mind–body intervention; rfi, reflective intervention; ssm, skills-based stress management

Table S5: Transformations and imputations applied to burnout outcome data.

Study (Author, year)	Outcome domain	Instrument	Time Point	Original reporting format	Imputation method
Dowers et al., 2017	EE/DP/RE/GB	MBI, ProQOL	Pre, Post	Median, IQR	Median and IQR converted to mean and SD assuming approximate normality.
Fraiman et al., 2022	EE/DP/RE	MBI	Post	Median	Post-intervention SD was imputed by substituting the corresponding baseline SD.
Gunasingam et al., 2015	EE/DP/RE	MBI	Pre, Post	Median, 95%CI	SD was derived from the reported mean, 95% CI, and sample size.
Huang et al., 2024	GB	ProQOL	Pre, Post	Median, IQR	Median and IQR converted to mean and SD assuming approximate normality.
Martins et al., 2011	EE/DP/RE	MBI	Pre, Post	Median	SD was imputed from pooled SDs of trials using the same instrument in a similar population.

Pre, baseline; Post, end of follow-up.

Abbreviations:

EE, emotional exhaustion; DP, depersonalization; RE, reduced efficacy; GB, global burnout; SD, standard deviation; CI, confidence interval; IQR, interquartile range; MBI, Maslach Burnout Inventory; ProQOL, Professional Quality of Life Scale.

Supplementary Methods: Study selection—use of active learning (ASReview).

After deduplication, during process of "Records screened" to "Reports sought for retrieval (**Figure 1**)," title/abstract screening was underwent using an active-learning workflow (ASReview LAB, version 1.6.3).[20] We uploaded the structured citation file (title, abstract, authors, year, journal/keywords) and seeded the model with one known relevant and three irrelevant records identified a priori. Unless otherwise noted, default settings were used. Two reviewers (Y.S. and K.T.) screened independently and blinded to each other's decisions; ASReview prioritized order of presentation only—final include/exclude (relevant/irrelevant) decisions were made by humans.

We pre-specified a stop rule: terminate when the reviewer encountered ≥ max (1% of the corpus, or 25) consecutive excludes.[21] To mitigate false-negative risk, we conducted a verification after stopping: confirmed that sentinel trials from prior reviews[7] were retrieved and included. Disagreements at any stage were resolved by consensus; full texts proceeded to dual independent assessment.

Apart from prioritization at the title/abstract stage, no automation tools were used; full-text screening, data extraction, risk-of-bias assessment, and synthesis were performed manually.
